# Supplementary material for: DNA methylation inhibitor attenuates polyglutamine‐induced neurodegeneration by regulating Hes5
Source: EMBO Mol Med. 2019 Apr 1;11(5):e8547. doi: 10.15252/emmm.201708547 (PMC6505579; doi:10.15252/emmm.201708547)
Supplement: Supplementary file 1 — Appendix [file EMMM-11-e8547-s001.pdf]

DNA methylation inhibitor attenuates polyglutamine-induced neurodegeneration by regulating Hes5

Naohide Kondo, Genki Tohnai, Kentaro Sahashi, Madoka Iida, Mayumi Kataoka, Hideaki Nakatsuji, Yutaka Tsutsumi, Atsushi Hashizume, Hiroaki Adachi, Haruki Koike, Keiko Shinjo, Yutaka Kondo, Gen Sobue, Masahisa Katsuno

Table of Contents

Appendix Figures S1 – S15
Appendix Tables S1 – S3

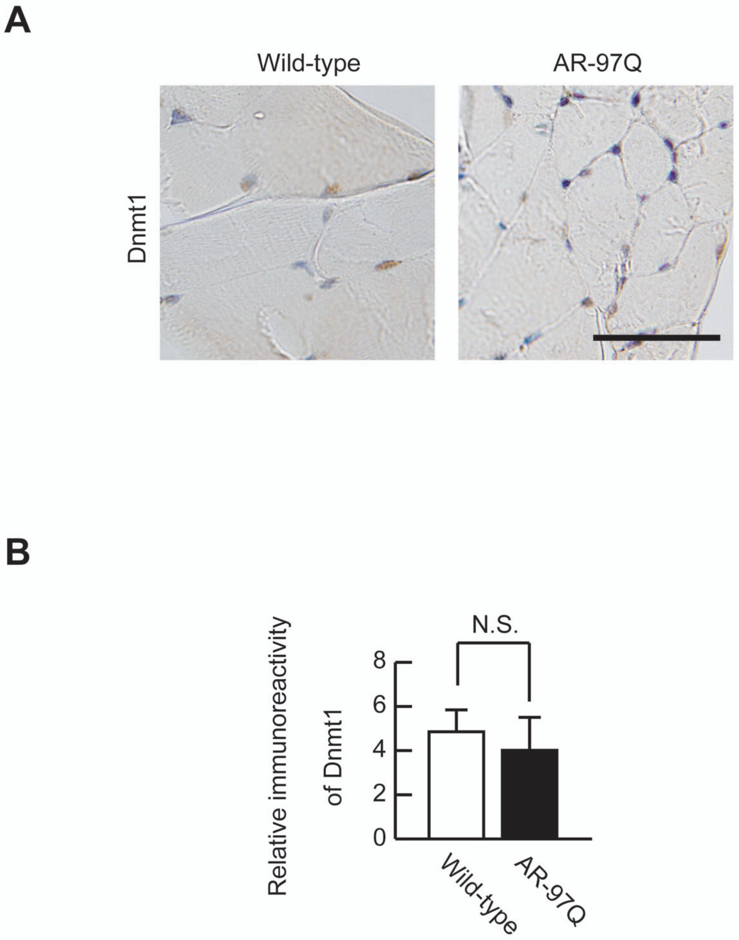

Appendix FigS1. (A) Representative immunohistochemistry images for Dnmt1 in the skeletal muscles of wild-type and AR-97Q mice. (B) Quantification of Dnmt1 immunoreactivity in the skeletal muscles of wild-type and AR-97Q mice (n = 3). Unpaired t-test. Error bars, s.e.m. (B). Scale bar, 20 μm (A). N.S.; not significant. The exact *P* value is in Appendix Table S3.

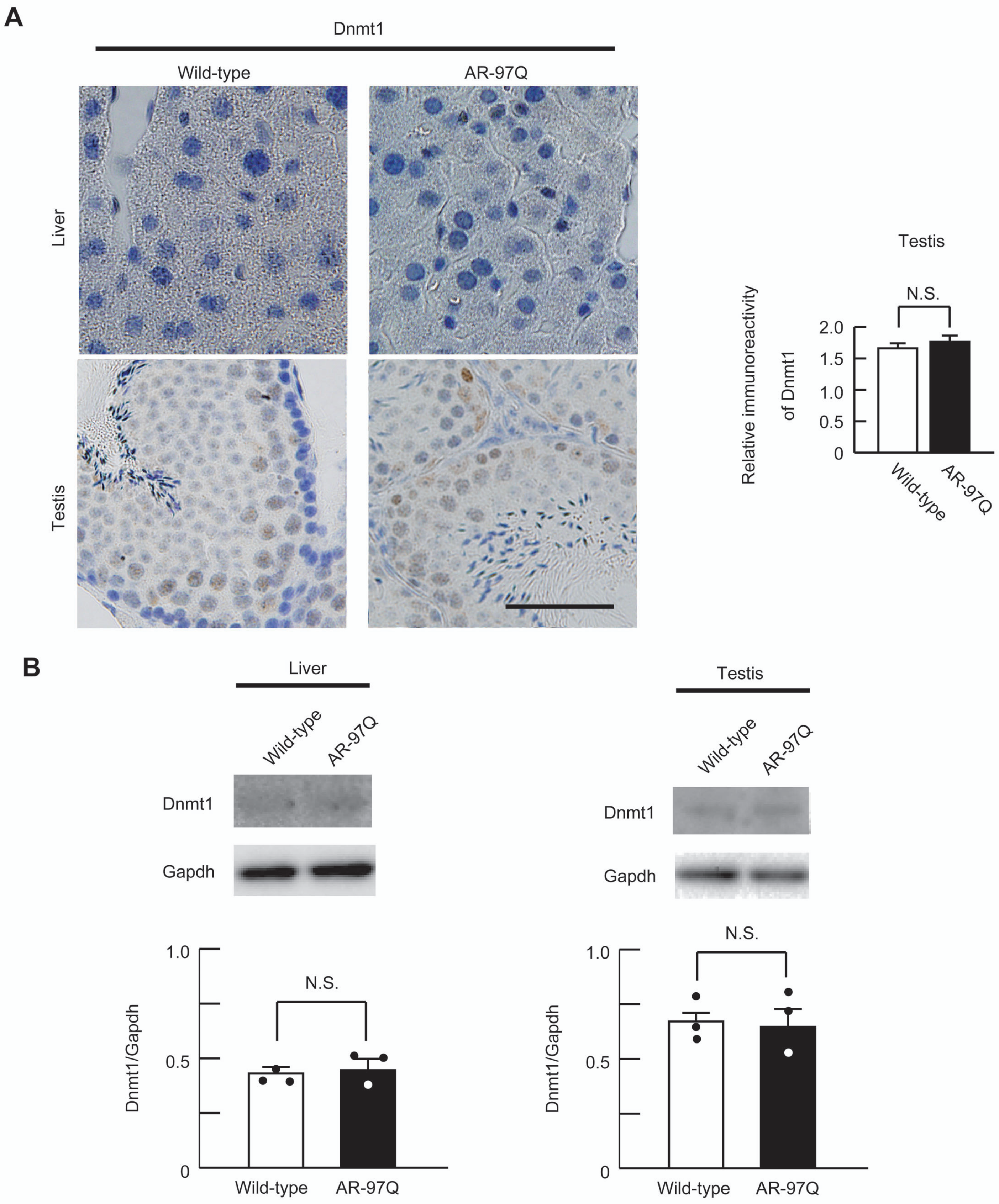

Appendix FigS2. Dnmt1 staining in the liver and testis of SBMA model mice.

(A) Representative images of Immunohistochemistry of the liver and the testis for Dnmt1 and relative immunoreactivity of Dnmt1 in the testis (n=3). (B) Representative bands and quantification of them of western blots for Dnmt1 of the liver and testis of wild-type and AR-97Q mice (n=3). Dnmt1 was rarely expressed in the liver both in wild-type and AR-97Q mice. Unpaired t-test. Error bars, s.e.m. (B). Scale bar, 20 μm (A). N.S.; not significant. The exact *P* value is in Appendix Table S3.

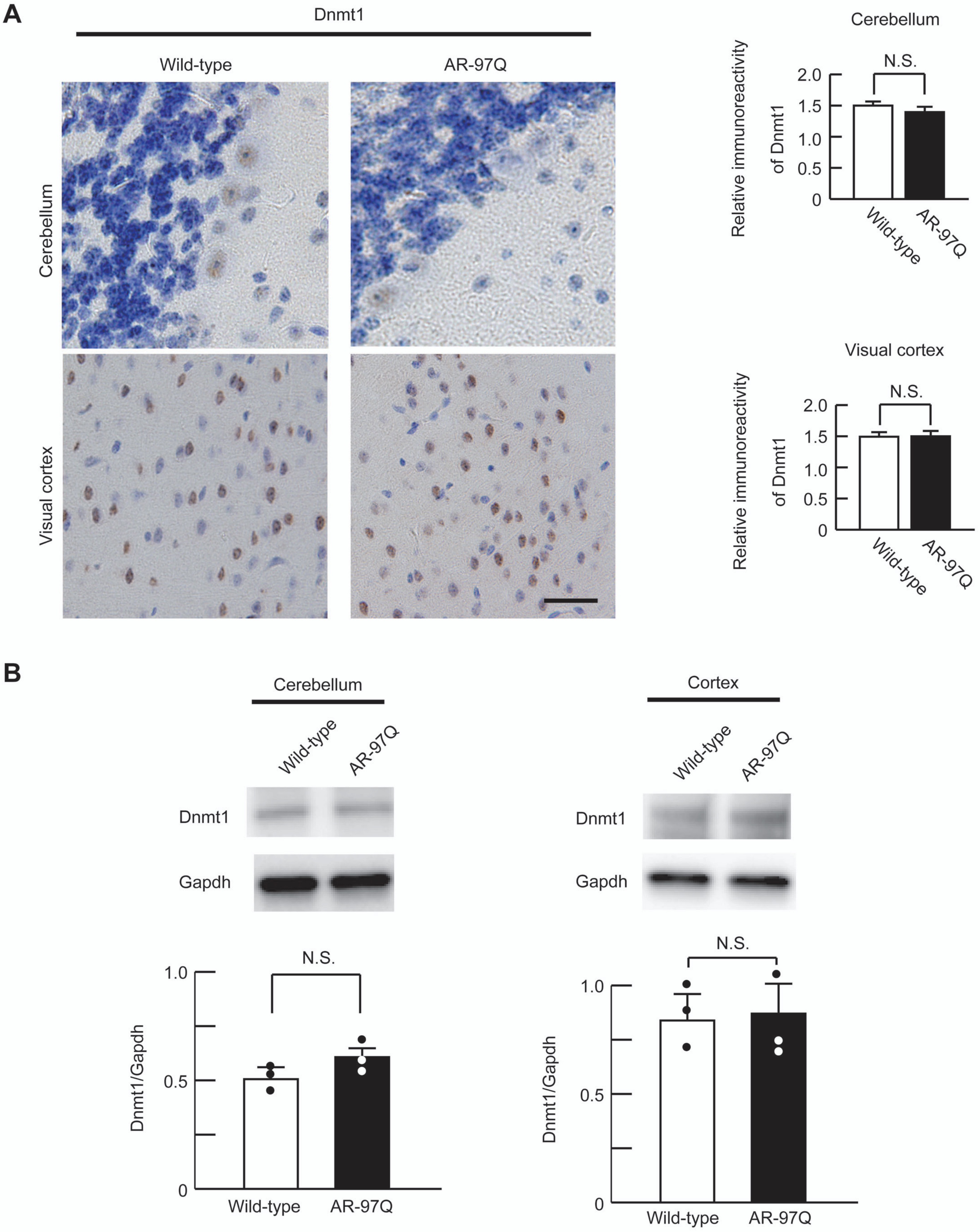

Appendix FigS3. Dnmt1 staining in the cerebellum and cefrebral cortex of SBMA model mice. (A) Representative images of Immunohistochemisty and quantified result of them for Dnmt1 (n=3). (B) Westenblots for Dnmt1 of the cerebellum and cerebral cortex of wild-type and AR-97Q mice (n=3). Unpaired t-test. Error bars, s.e.m. (A, B). Scale bar, 20 µm (A). N.S.; not significant. The exact *P* value is in Appendix Table S3.

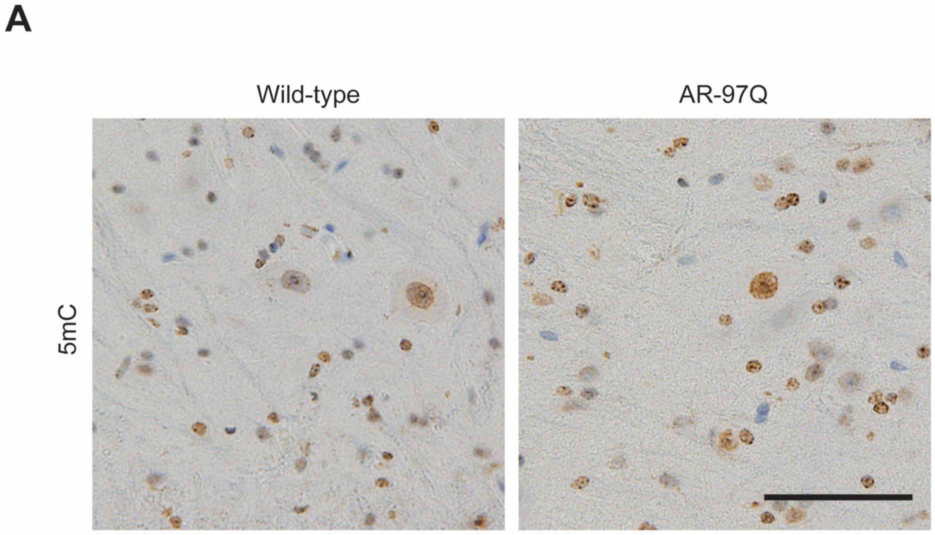

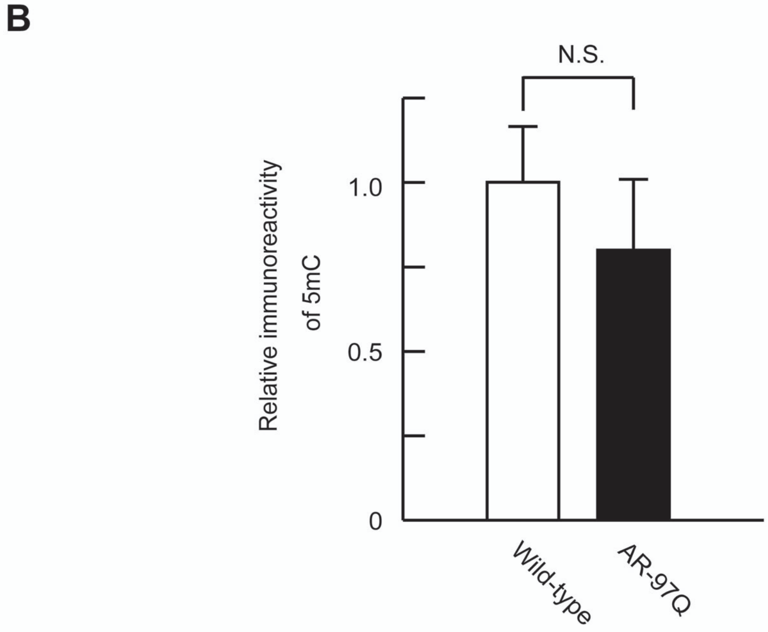

Appendix FigS4. Immunostaining of 5-methylcytosine (5mC) in the spinal motor neurons of SBMA model mice. (A) Representative images of immunohistochemistry for 5mC. (B) Quantitative analysis of immunoreactivity for 5mC in the motor neurons of spinal anterior horn of wild-type and AR-97Q mice(n = 3 per group). Unpaired t-test. Error bars, s.e.m. (B). Scale bar 20μm (A). N.S.; not significant. The exact *P* value is in Appendix Table S3.

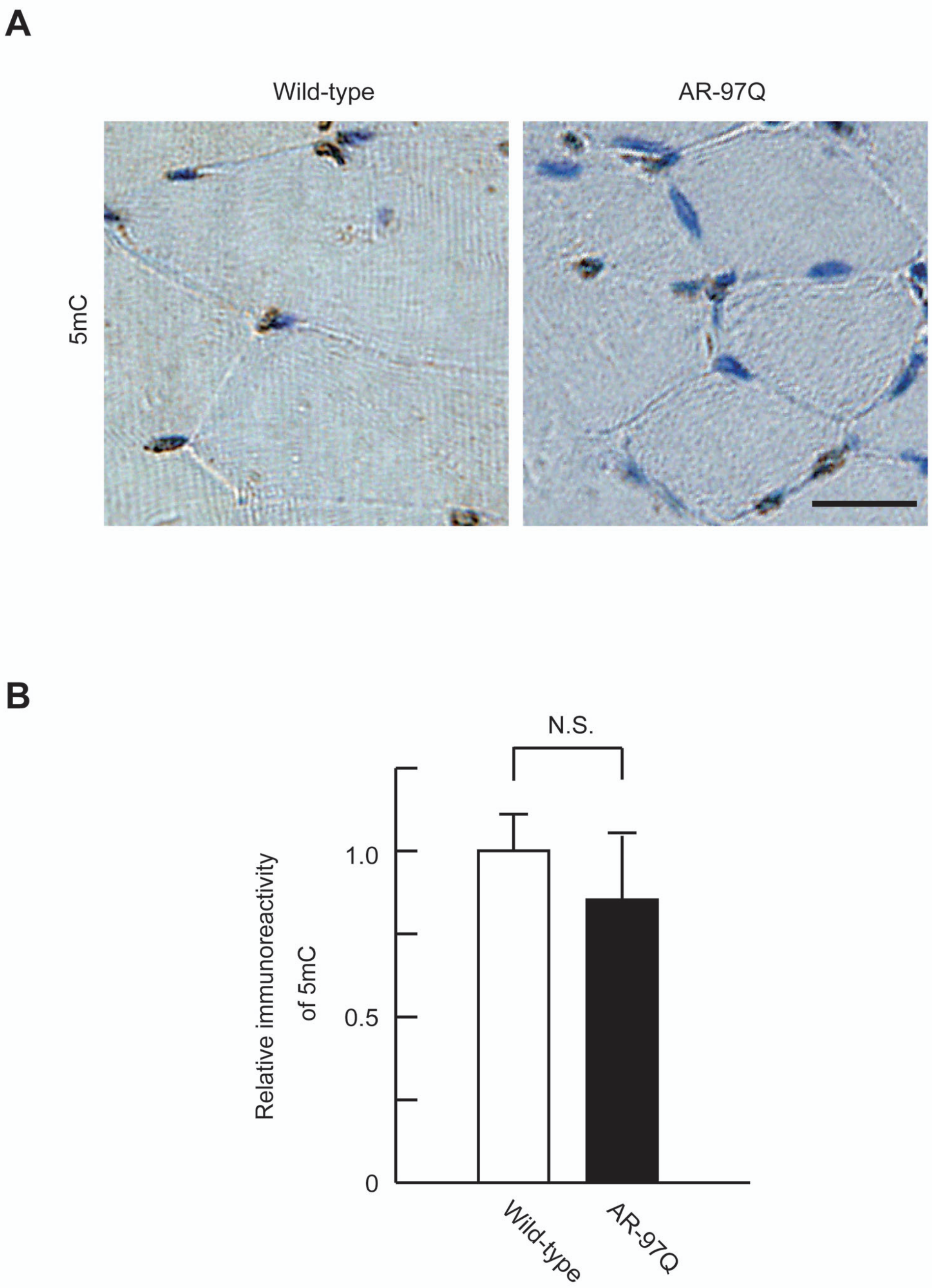

Appendix FigS5. 5-methylcytosine (5mC) immunoreactivity in the skeletal muscle of mice model of SBMA. (A) Representative images of immunohistochemistry of skeletal muscle for 5mC. (B) Quantitative analysis of immunoreactivity for 5mC in the tissue (n = 3 per group). Unpaired t-test. Error bars, s.e.m. (B). Scale bar 20μm (A). N.S.; not significant. The exact *P* value is in Appendix Table S3.

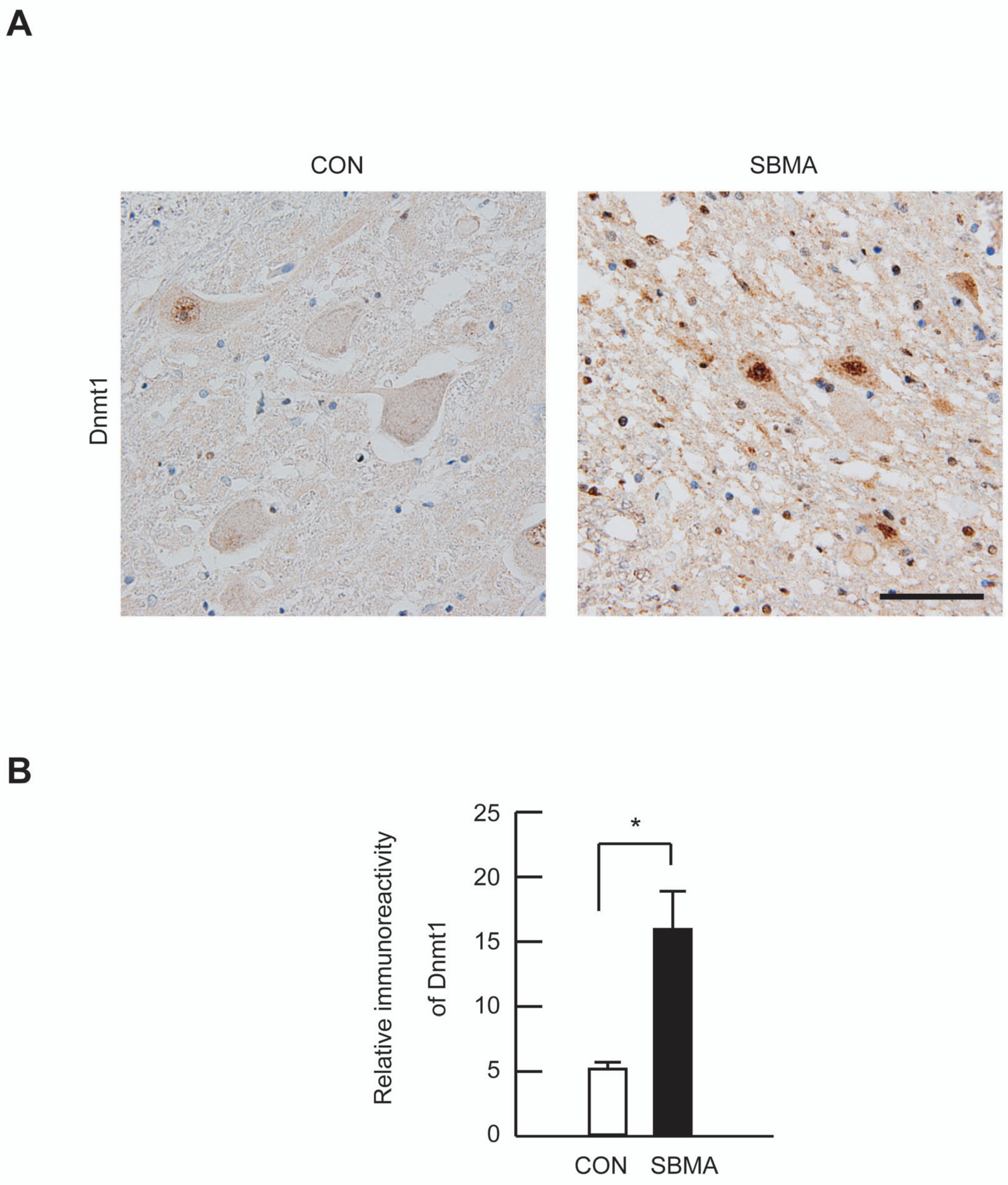

Appendix FigS6. Dnmt1 staining of the spinal motor neuron of patients with SBMA. Representative images of immunohistochemisty for Dnmt1 in the spinal anterior horns of subjects with SBMA and control patients (CON). (B) Quantitative analysis of immunoreactivity for Dnmt1 (*n* = 3 per group). Unpaired t-test. Error bars, s.e.m. (B). Scale bar 20μm (A). **P* < 0.05. The exact *P* value is in Appendix Table S3.

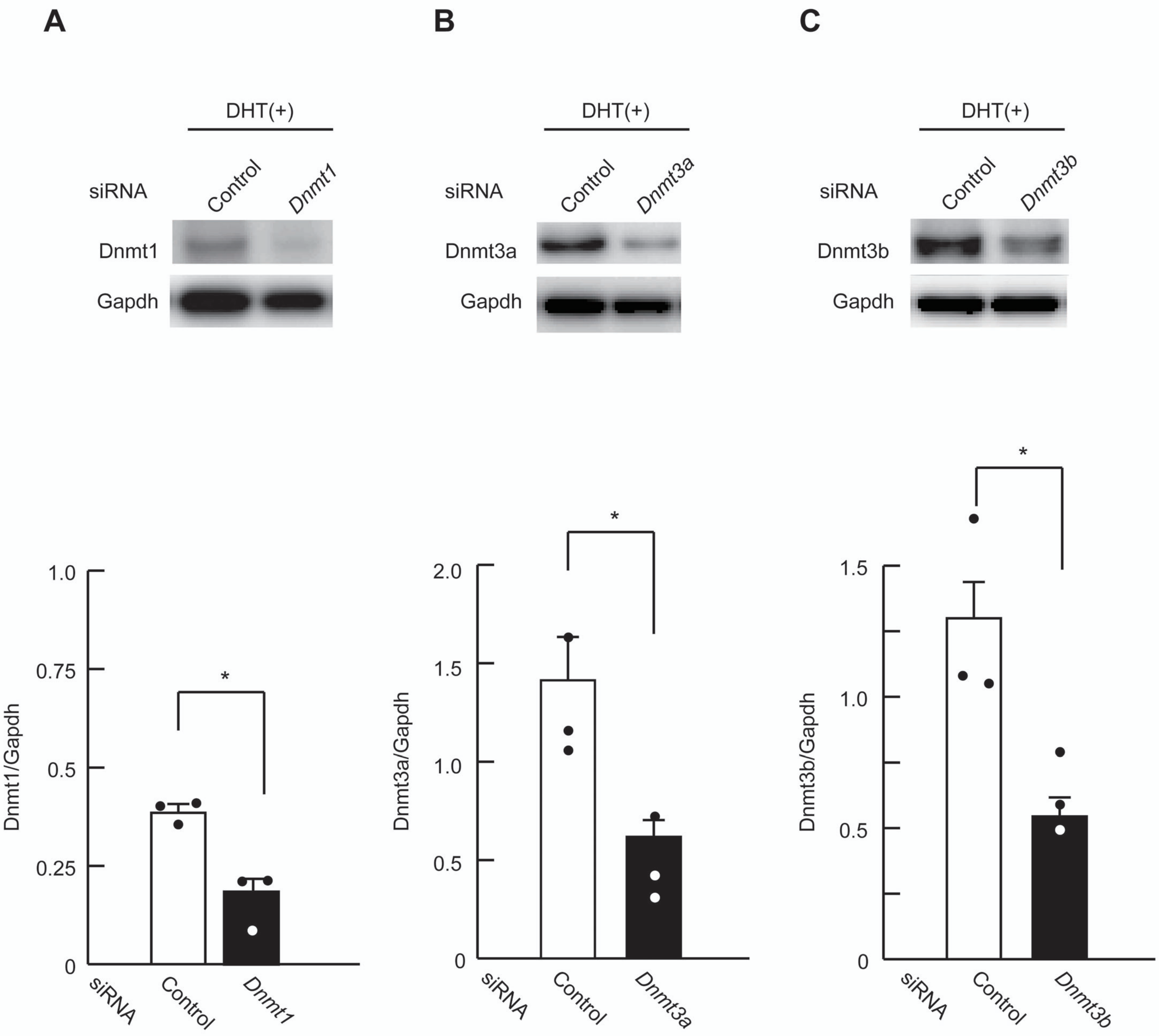

Appendix FigS7. Efficacy of siRNA-mediated knockdown of *Dnmts* in neuronal cellular model of SBMA. (A-C) Representative bands and quantitative analyses of immunoblotting of NSC34 cells expressing AR-97Q treated with siRNA for *Dnmt1*(A), *Dnmt3a*(B) or *Dnmt3b*(C) (n = 3). Unpaired t-test. Error bars, s.e.m. *P < 0.05. The exact P value is in Appendix Table S3.

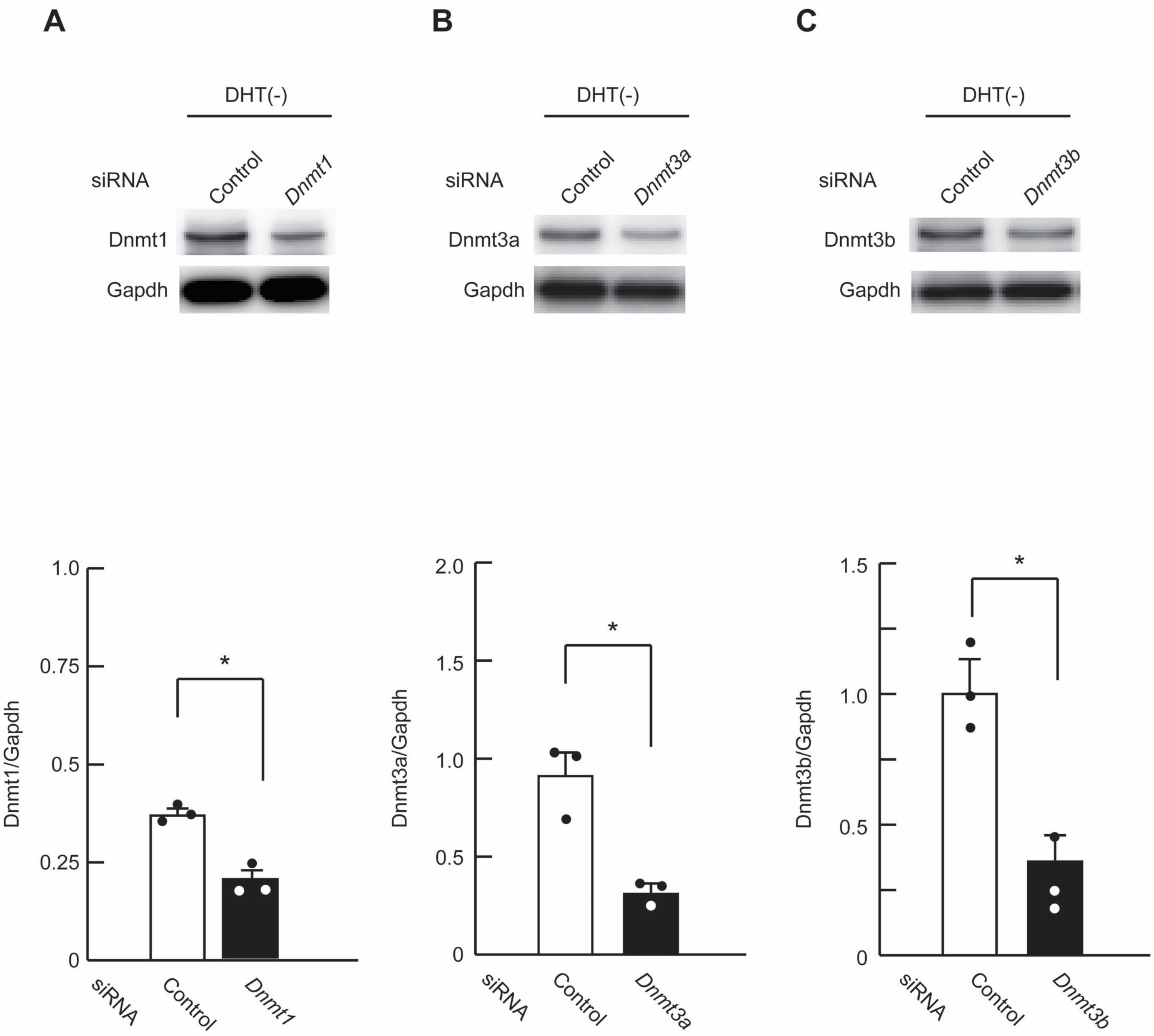

Appendix FigS8. Efficacy of siRNA-mediated knockdown of *Dnmts* in DHT-untreated NSC97Q cells. (A-C) Representative bands and quantitative analyses of immunoblotting of NSC34 cells expressing AR-97Q treated with siRNA for *Dnmt1* (A), *Dnmt3a*(B) or *Dnmt3b*(C) (n = 3) without DHT. Unpaired t-test. Error bars, s.e.m. *$P < 0.05$. The exact P value is in Appendix Table S3.

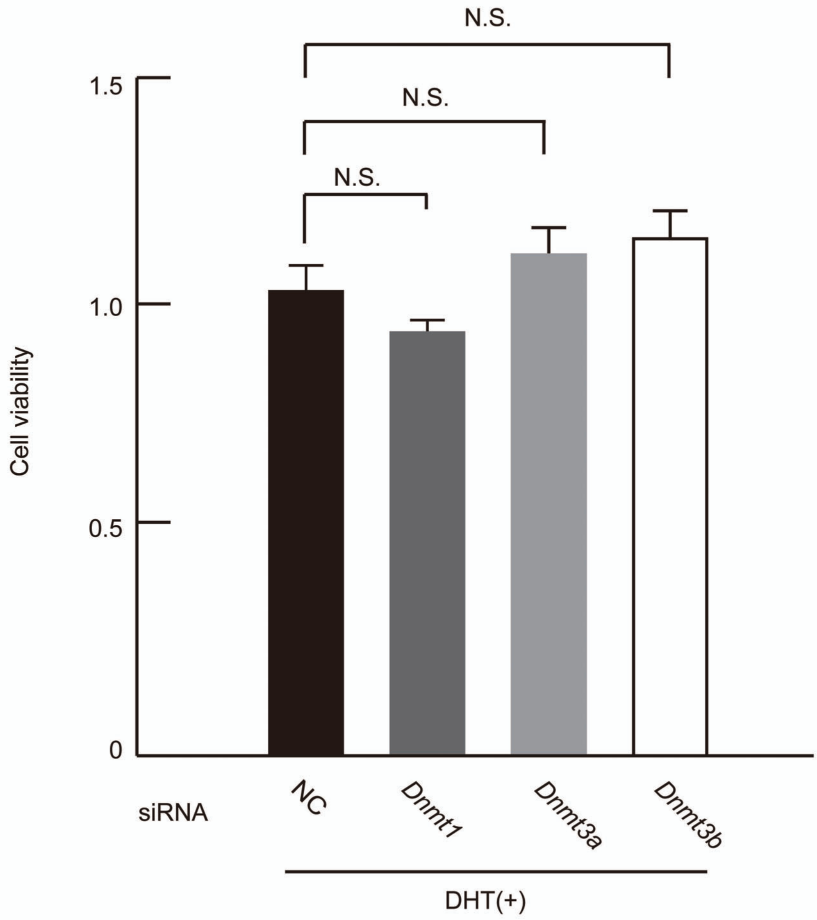

Appendix FigS9. WST-8 assay of siRNA-mediated knockdown of *Dnmts* in DHT-treated NSC24Q cells. Cell viability was not altered by Dnmts knockdown in the NSC34 cells expressing AR-24Q treated with siRNA for *Dnmt1* with DHT (n = 3). Unpaired t-test. Error bars, N.S. not significant. The exact P value is in Appendix Table S3.

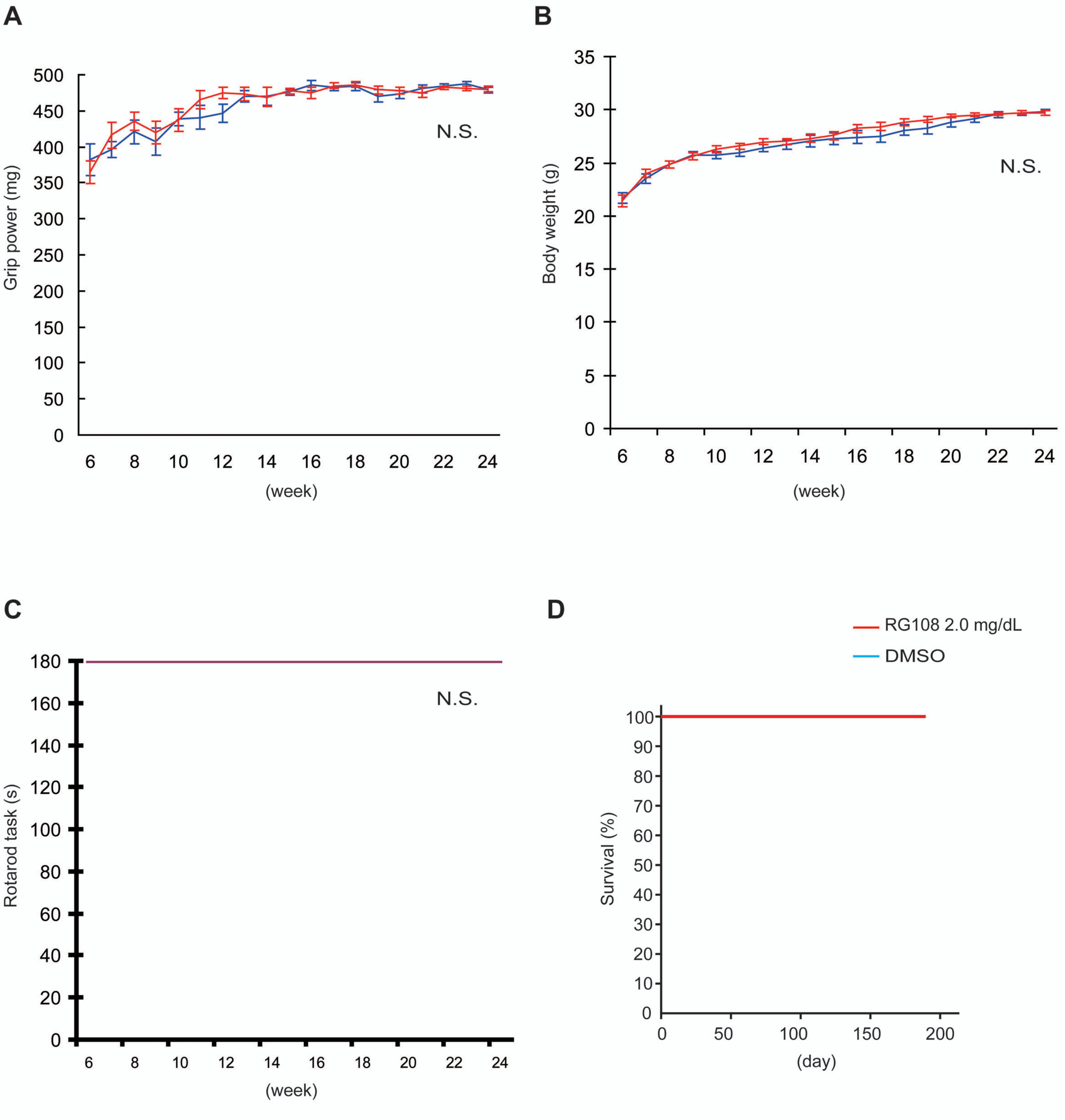

Appendix FigS10. RG108 treatment for wild-type mice. (A-D) Grip power (A), body weight (B), rotarod performance (C) and survival rate (Kaplan-Meier analysis and log-rank test) (D) of wild-type mice treated with DMSO or without RG108 (2.0mg/dL, n = 10). All parameters were not alterd in wild-type mice treated with RG108 at the dose of 2.0mg/dL compared with those treated with saline. Two-way ANOVA with Tukey test (A-C). Error bars, s.e.m (A,B). N.S.; not significant. The exact *P* value is in Appendix Table S3.

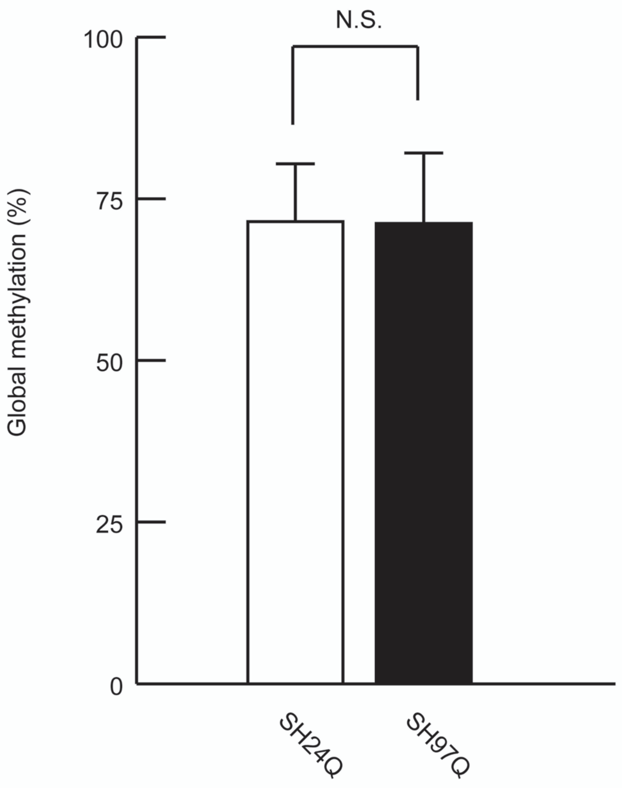

Appendix FigS11. Global DNA methylation rate of SBMA model cells. Human Methylation 450K array analysis revealed that global DNA methylation rate was not significantly different between SH-SY5Y cells stably expressing AR-24Q (SH24Q) and those expressing AR-97Q (SH97Q) (n = 3). Unpaired t-test. Error bars, s.e.m. N.S.; not significant. The exact P value is in Appendix Table S3.

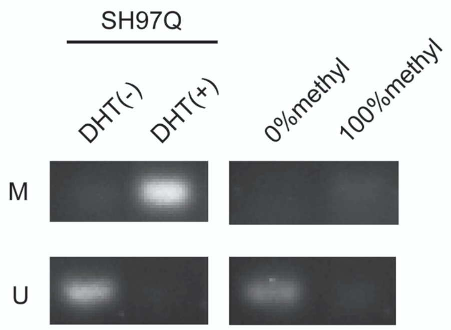

Appendix FigS12. Methylation-specific PCR analysis (MSP) of SH-SY5Y cells stably expressing AR-97Q (SH97Q) treated with or without DHT. Methylation of the HES5 promoter CpG island in DHT-treated SH97Q cells was higher than that of DHT-untreated SH97Q cells.

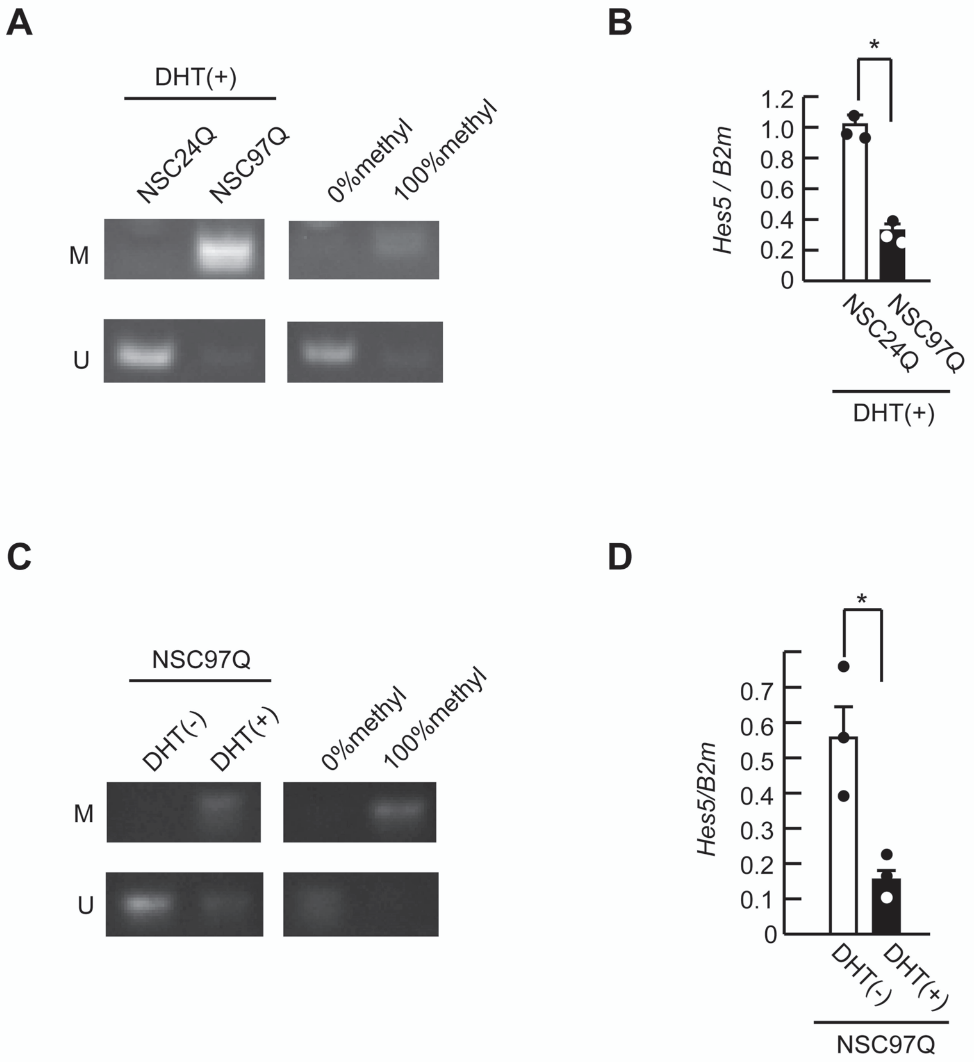

Appendix FigS13. Methylation-specific PCR analysis (MSP) and RT-qPCR for *Hes5*. (A) MSP for Hes5 in NSC24Q and NSC97Q cells treated with DHT. Hyper-methylation was observed in the promoter CpG island in DHT-treated NSC97Q. (B) *Hes5* mRNA level was reduced in DHT-treated NSC97Q compared with NSC24Q (n=3). (C) MSP for *Hes5* in NSC97Q with or without DHT. DHT-treated NSC97Q cells have hyper-methylation in the CpG island of Hes5 promoter. (D) DHT treatment induced *Hes5* reduction in NSC97Q cells by RT-qPCR analysis (n=3). Unpaired t-test. Error bars, s.e.m. *P < 0.05. The exact P value is in Appendix Table S3.

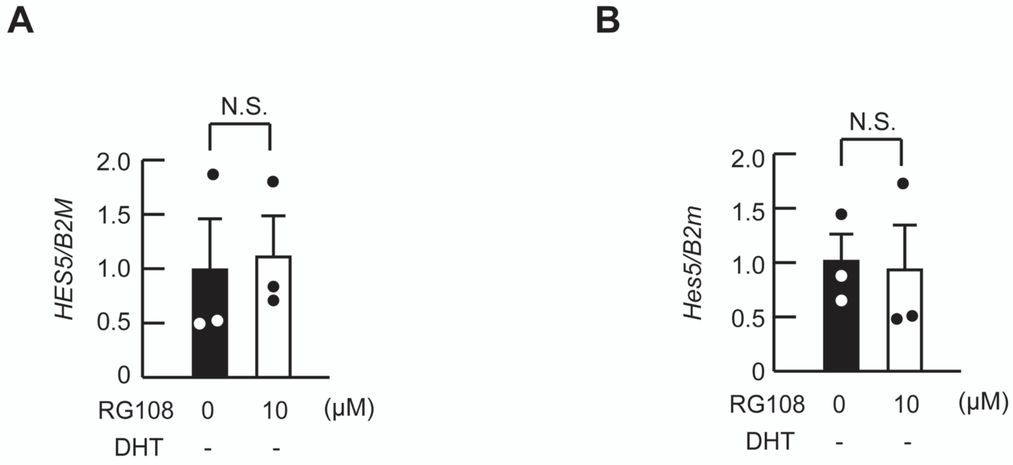

Appendix FigS14. RG108 had no significant effect to the relative mRNA level of *Hes5*.

(A) *Hes5* expression level was not altered by RG108 in DHT-untreated SH97Q cells (n=3).

(B) RG108 had no influence on *Hes5* expression in NSC97Q cells without DHT (n=3).

Unpaired t-test. Error bars, s.e.m. (A,B). N.S.; not significant. The exact *P* value is in Appendix Table S3.

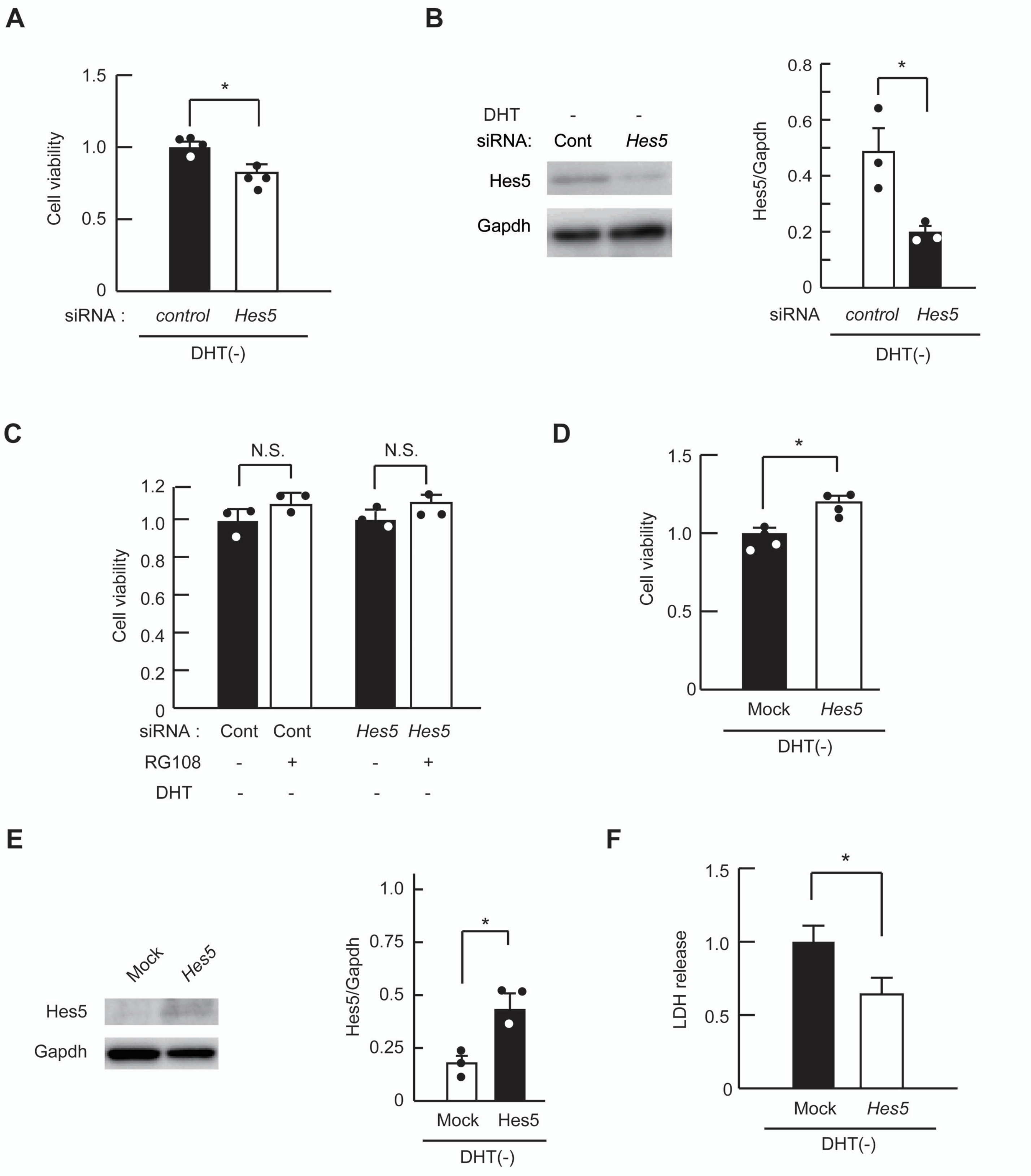

Appendix FigS15. (A, B) siRNA-mediatd knockdown of *Hes5* reduced the cell viability of DHT-untreated NSC97Q cells (n=3). (C) WST-8 assay revealed that the cell viability was not alterd by RG108 treatment in DHT-untreated NSC97Q cells (n=3). (D, E, F) Hes5 over -expression had a positive effect to NSC97Q cells without DHT. (n=3(D,E), n=6 (F).) Unpaired t-test. Error bars, s.e.m. *$P < 0.05$. N.S.; not significant. The exact *P* value is in Appendix Table S3.

Appendix Table S1. List of genes which have hyper-methylation in the promoter region of SH-97Q cells with DHT compared with SH-24Q cells with DHT.

ID	GENE_SYMBOL	p_value	q_value_BH	LogRatio
NM_004358	CDC25B	1.44E-06	0.000912	3.829732
NM_021872	CDC25B	1.44E-06	0.000912	3.829732
NM_021873	CDC25B	1.44E-06	0.000912	3.829732
NM_001496	GFRA3	1.07E-06	0.000843	3.77169
NM_000905	NPY	8.88E-07	0.000828	3.416165
NM_001010926	HES5	3.89E-07	0.000719	3.117869
NM_002980	SCTR	2.71E-06	0.001295	3.022521
NR_029373	LEF1-AS1	1.2E-06	0.000908	2.894203
NM_033122	CABS1	0.000315	0.010804	2.818629
NM_001145545	C16orf82	1.76E-06	0.001011	2.78421
NM_000953	PTGDR	2.12E-07	0.000672	2.675928
NM_033258	GNG8	2.14E-06	0.001147	2.669694
NM_144650	ADHFE1	0.000325	0.010947	2.619538
NM_001437	ESR2	2.75E-05	0.003362	2.579769
NM_001040275	ESR2	2.75E-05	0.003362	2.579769
NM_001040276	ESR2	2.75E-05	0.003362	2.579769
NM_018556	SIRPG	2.45E-05	0.003296	2.573056
NM_080816	SIRPG	2.45E-05	0.003296	2.573056
NM_001039508	SIRPG	2.45E-05	0.003296	2.573056
NM_152628	SNX31	5.13E-05	0.004515	2.554422
NM_015472	WWTR1	1.27E-05	0.002773	2.553708
NM_001168280	WWTR1	1.27E-05	0.002773	2.553708
NM_181539	KRT26	5.95E-07	0.000719	2.549864
NM_058186	FAM3B	6.5E-08	0.000672	2.545396
NM_206964	FAM3B	6.5E-08	0.000672	2.545396
NM_178169	RASSF3	6.34E-05	0.004859	2.514891
NM_021250	LILRA5	1.96E-05	0.003024	2.469218
NM_181879	LILRA5	1.96E-05	0.003024	2.469218
NM_181985	LILRA5	1.96E-05	0.003024	2.469218
NM_181986	LILRA5	1.96E-05	0.003024	2.469218
NM_001004757	OR51Q1	9.71E-05	0.006149	2.467823
NR_029522	MIR105-2	7.7E-06	0.002228	2.464793
NM_138780	SYTL5	0.000102	0.006276	2.441371
NM_001163335	SYTL5	0.000102	0.006276	2.441371
NM_207517	ADAMTSL3	3.47E-05	0.003712	2.408943
NM_173674	DCBLD1	1.11E-07	0.000672	2.382309
NM_138970	NRXN3	0.000145	0.007278	2.371849
NM_001105250	NRXN3	0.000145	0.007278	2.371849

Appendix Table S2. List of genes which have hypo-rmethylation in the promoter region of SH-97Q cells with DHT compared with SH-24Q cells with DHT.

ID	GENE_SYMBOL	p_value	q_value_BH	LogRatio
NM_080284	ABCA6	0.002086	0.031398	-2.8516
NM_000624	SERPINA5	6.12E-06	0.001989	-2.7119
NM_002351	SH2D1A	0.000374	0.011693	-2.64043
NM_001114937	SH2D1A	0.000374	0.011693	-2.64043
NM_002705	PPL	0.000933	0.019526	-2.58601
NM_013327	PARVB	2.95E-05	0.003478	-2.56105
NM_000828	GRIA3	0.000207	0.008455	-2.49857
NM_007325	GRIA3	0.000207	0.008455	-2.49857
NR_026833	LOC400940	0.000173	0.007787	-2.41081
NM_175060	CLEC14A	0.000281	0.010123	-2.40878
NM_145038	CCDC164	0.000145	0.007278	-2.38714
NM_001005853	OR6B2	9.59E-05	0.006115	-2.37879

Appendix TableS3. Exact p-values of each figure.

Figure	Compared group	p-value	Indication
Fig. 1B	WT vs 24Q vs 97Q (Dnmt1)	0.0007	*
Fig. 1B	WT vs 24Q vs 97Q (Dnmt3a)	0.5654	
Fig. 1B	WT vs 24Q vs 97Q (Dnmt3b)	0.4128	
Fig. 1C	WT vs 24Q vs 97Q (Dnmt1)	0.0005	*
Fig. 1C	WT vs 24Q vs 97Q (Dnmt3a)	0.5757	
Fig. 1C	WT vs 24Q vs 97Q (Dnmt3b)	0.5192	
Fig. 1E	WT vs 24Q vs 97Q (Dnmt1)	<0.0001	*
Fig. 1E	WT vs 24Q vs 97Q (Dnmt3a)	0.9843	
Fig. 1E	WT vs 24Q vs 97Q (Dnmt3b)	0.1270	
Fig. 1H	WT vs 97Q (Dnmt1)	0.9789	
Fig. 2A	NSC24 vs NSC97 (Dnmt1)	0.0377	*
Fig. 2A	NSC24 vs NSC97 (Dnmt3a)	0.9175	
Fig. 2A	NSC24 vs NSC97 (Dnmt3b)	0.3103	
Fig. 2B	NSC24 vs NSC97 (Dnmt1) RNA	0.0053	*
Fig. 2C	NSC97 DHT(-) vs DHT(+) (Dnmt1)	0.0028	*
Fig. 2C	NSC97 DHT(-) vs DHT(+) (Dnmt3a)	0.9730	
Fig. 2C	NSC97 DHT(-) vs DHT(+) (Dnmt3b)	0.6193	
Fig. 2D	NSC97 DHT(-) vs DHT(+) (Dnmt1) RNA	0.0029	*
Fig. 2E	NC vs siRNA_Dnmt1	0.0001	*
Fig. 2E	NC vs siRNA_Dnmt3a	0.5024	
Fig. 2E	NC vs siRNA_Dnmt3b	0.1441	
Fig. 2F	NC vs siRNA_Dnmt1	0.6249	
Fig. 2F	NC vs siRNA_Dnmt3a	0.0804	
Fig. 2F	NC vs siRNA_Dnmt3b	0.2444	
Fig. 2G	RG0 vs RG0.1	0.1513	
Fig. 2G	RG0 vs RG1	0.0490	*
Fig. 2G	RG0 vs RG10	0.0021	*
Fig. 2I	RG0 vs RG0.1	0.1630	

Fig. 2I	RG0 vs RG1	0.1532	
Fig. 2I	RG0 vs RG10	0.5496	
Fig. 2J	RG0 vs RG0.1	0.0820	
Fig. 2J	RG0 vs RG1	0.1534	
Fig. 2J	RG0 vs RG10	0.7373	
Fig. 3A	Grip DMSO vs RG0.5	<0.001	*
	Grip DMSO vs RG.0	<0.001	*
Fig. 3B	BW DMSO vs RG0.5	<0.001	*
	BW DMSO vs RG2.0	<0.001	*
Fig. 3C	Rotarod DMSO vs RG0.5	<0.001	*
	Rotarod DMSO vs RG2.0	<0.001	*
Fig. 3D	Survival rate DMSO vs RG0.5	<0.001	*
Fig. 3G	DMSO vs RG108 (Dnmt1) WB	0.0061	*
Fig. 3I	DMSO vs RG108 (Dnmt1) IHC	0.0002	*
Fig. 4B	1C2 DMSO vs RG108	0.7271	
Fig. 4D	AR DMSO vs RG108	0.7149	
Fig. 4E	humanAR DMSO vs RG108	0.0764	
Fig. 4G	Chat DMSO vs RG108	0.0214	*
Fig. 4I	Chat DMSO vs RG108	0.0091	*
Fig. 5B	SH24 vs SH97 (Dnmt1)	0.0269	*
Fig. 5B	SH24 vs SH97 (Dnmt3a)	0.3438	
Fig. 5B	SH24 vs SH97 (Dnmt3b)	0.6474	
Fig. 5D	SH97 DHT(-) vs DHT(+) (Dnmt1)	0.0483	*
Fig. 5D	SH97 DHT(-) vs DHT(+) (Dnmt3a)	0.9159	
Fig. 5D	SH97 DHT(-) vs DHT(+) (Dnmt3b)	0.2113	
Fig. 5F	CDC25B SH24 vs SH97	0.2097	
Fig. 5F	GFRA3 SH24 vs SH97	0.4430	
Fig. 5F	NPY SH24 vs SH97	0.1060	
Fig. 5F	HES5 SH24 vs SH97	<0.0001	*
Fig. 5F	SCTR SH24 vs SH97	0.6513	

Fig. 5F	LEF1 SH24 vs SH97	0.9674	
Fig. 5F	CABS1 SH24 vs SH97	0.4914	
Fig. 5G	SH97 DHT(-) vs DHT(+) (Hes5)	0.0445	*
Fig. 5I	WT vs 24Q vs 97Q (Hes5)	<0.0001	*
Fig. 5J	WT vs 24Q vs 97Q (Hes5)	<0.0001	*
Fig. 6B	RG108 0 vs 10 (HES5)	0.0006	*
Fig. 6D	RG108 0 vs 10 (Hes5)	0.0014	*
Fig. 6E	DMSO vs RG108 (Hes5)	0.0499	*
Fig. 6G	DMSO vs RG108 (Hes5)	<0.0001	*
Fig. 7A	NC vs siHes5	0.0330	*
Fig. 7B	NC vs siRNA_Hes5 (Hes5)	0.0151	*
Fig. 7C	RG (–) vs RG (+)	0.0257	*
Fig. 7C	siHes5_RG (–) vs RG (+)	0.2920	
Fig. 7D	Mock vs Hes5	0.0067	*
Fig. 7E	Mock vs Hes5	0.0032	*
Fig. 7F	Mock vs Hes5	0.0183	*
Fig. 7H	Mock vs Hes5	0.4384	
Fig. 8B	pSmad2 cont vs siHes5	0.0018	*
Fig. 8B	Smad2 cont vs siHes5	0.1061	
Fig. 8B	pIkBα cont vs siHes5	0.8400	
Fig. 8B	Hsf1 cont vs siHes5	0.9283	
Fig. 8C	cont vs siHes5	0.0020	*
Fig. 8D	pSmad2 DHT(-) vs DHT(+)	0.0470	*
Fig. 8D	Smad2 DHT(-) vs DHT(+)	0.7806	
Fig. 8E	Hes5 cont vs Hes5	0.0139	*
Fig. 8E	pSmad2 cont vs Hes5	0.0006	*
Fig. 8E	Smad2 cont vs Hes5	0.3902	
Fig. 8F	Hes5 AR24Q vs AR97Q	0.0028	*
Fig. 8F	pSmad2 AR24Q vs AR97Q	0.0084	*
Fig. 8F	Smad2 AR24Q vs AR97Q	0.7467	
Fig. 8G	Hes5 AR24Q vs AR97Q	0.0476	*
Fig. 8H	Hes5 AR24Q vs AR97Q	0.0107	*
FigS1B	Wild-type vs AR97Q	0.4347	
FigS2A	Wild-type vs AR97Q	0.1374	

FigS2B	Wild-type vs AR97Q	0.8150	
FigS2B	Wild-type vs AR97Q	0.8267	
FigS3A	Wild-type vs AR97Q	0.3826	
FigS3A	Wild-type vs AR97Q	0.8472	
FigS3B	Wild-type vs AR97Q	0.1368	
FigS3B	Wild-type vs AR97Q	0.9618	
FigS4B	5mC Wild-type vs AR97Q	0.6467	
FigS5	5mC Wild-type vs AR97Q	0.2588	
FigS6B	DNMT1 CON vs SBMA	<0.0001	*
FigS7A	control vs siDnmt1	0.0018	*
FigS7B	control vs siDnmt3a	0.0118	*
FigS7C	control vs siDnmt3b	0.0339	*
FigS8A	control vs siDnmt1	0.0015	*
FigS8B	control vs siDnmt3a	0.0080	*
FigS8C	control vs siDnmt3b	0.0088	*
FigS9	NC vs siDnmt1	0.1634	
FigS9	NC vs siDnmt3a	0.3420	
FigS9	NC vs Dnmt3b	0.0577	
FigS13B	NSC 24Q vs 97Q	0.0007	*
FigS13D	NSC97 DHT(-) vs DHT(+)	0.0283	*
FigS14A	SH97DHT(-) RG0 vs RG10	0.8576	
FigS14B	NSC97DHT(-) RG0 vs RG10	0.8342	
FigS15A	DHT(-) con vs siHes5	0.0153	*
FigS15B	DHT(-) con vs siHes5	0.0464	*
FigS15C	Cont RG(-) vs RG(+)	0.1212	
FigS15C	siHes5 RG(-) vs RG(+)	0.0596	
FigS15D	Mock vs Hes5	0.0156	*
FigS15E	Mock vs Hes5	0.0228	*
FigS15F	Mock vs Hes5	0.0145	*
