# Supplementary material for: DNA methylation inhibitor attenuates polyglutamine‐induced neurodegeneration by regulating Hes5
Source: EMBO Mol Med. 2019 Apr 1;11(5):e8547. doi: 10.15252/emmm.201708547 (PMC6505579; doi:10.15252/emmm.201708547)

AppendixFigS3

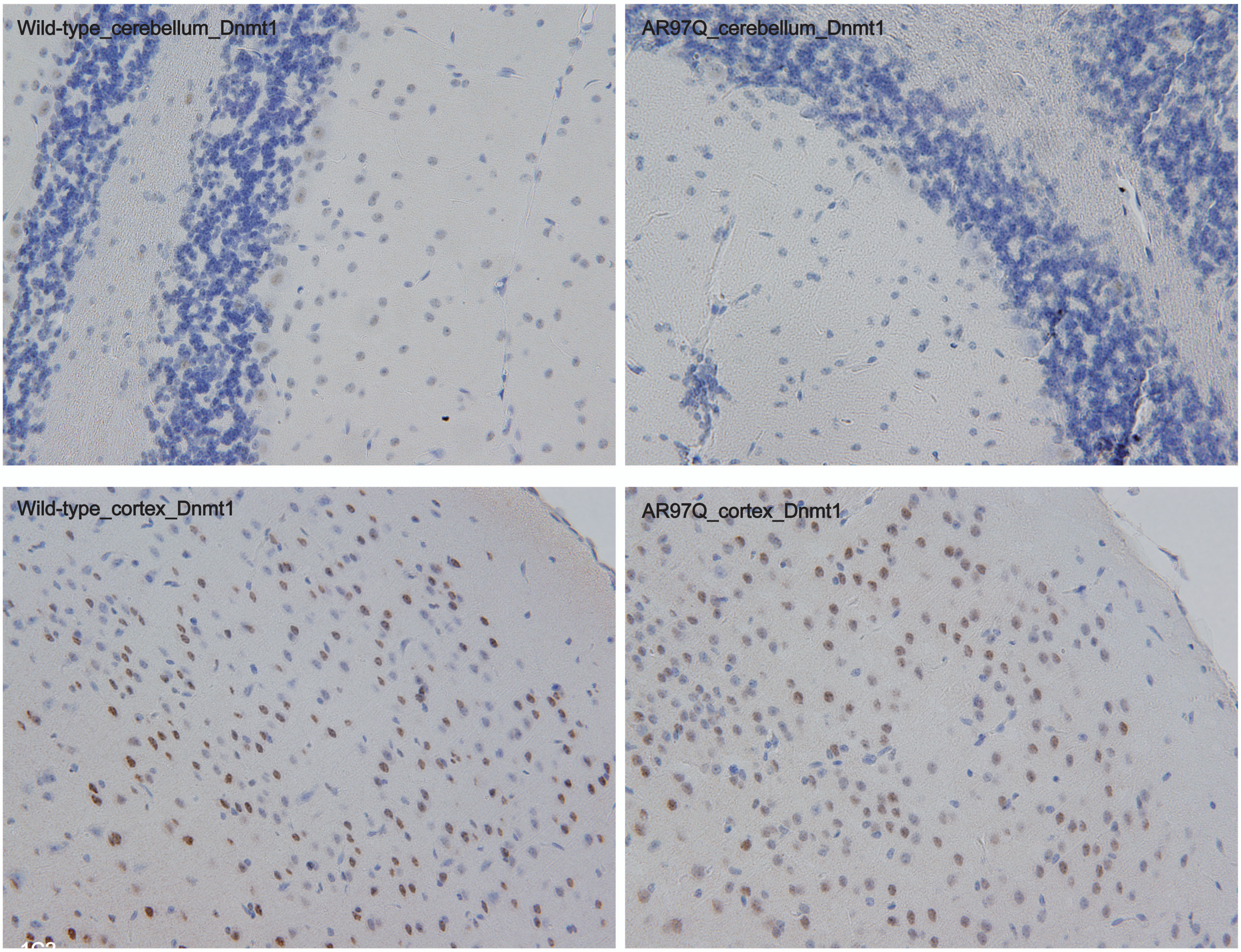

AppendixFigS3

AppendixFigS3B_Dnmt1

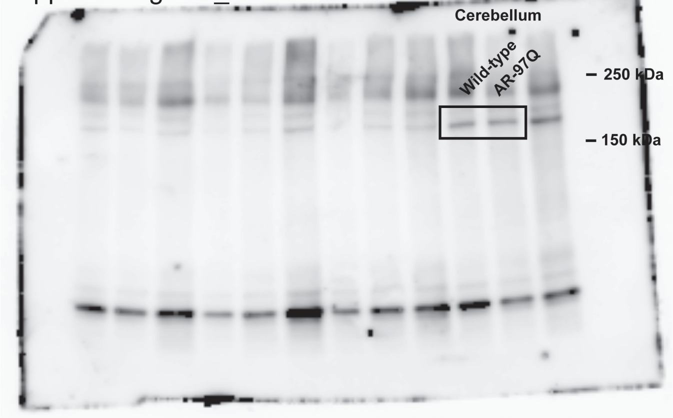

AppendixFigS3B_Gapdh

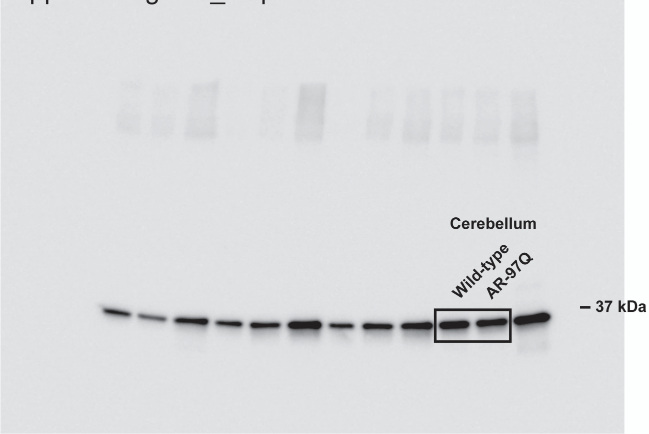

AppendixFigS3B_Dnmt1

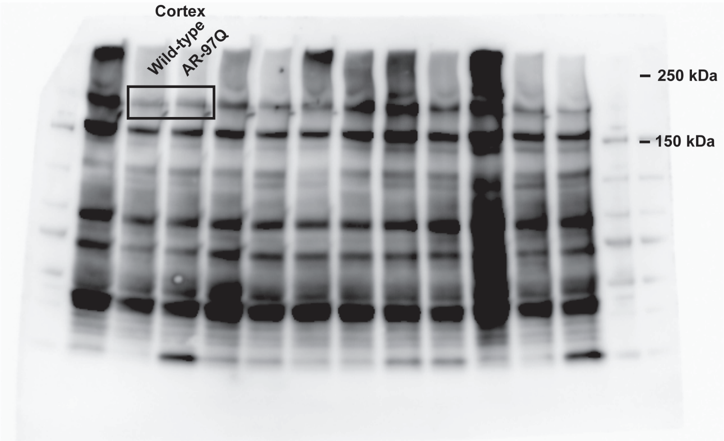

AppendixFigS3B_Gapdh

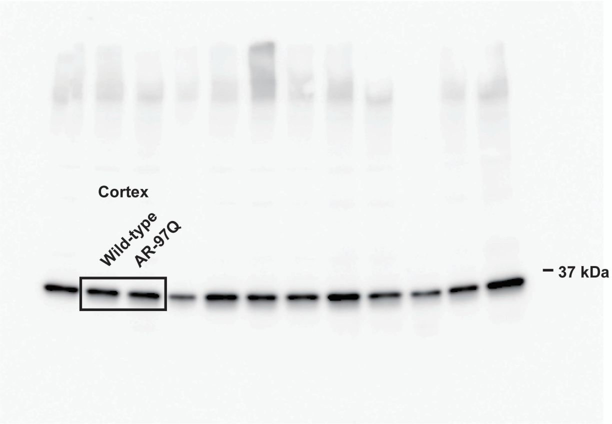

Supplement: Supplementary file 2 — Source Data for Appendix [file EMMM-11-e8547-s010.zip › SourceData_for_Appendix_Figures/SourceData_for_AppendixFigS3_1200DPI.pdf]
