# Supplementary material for: DNA methylation inhibitor attenuates polyglutamine‐induced neurodegeneration by regulating Hes5
Source: EMBO Mol Med. 2019 Apr 1;11(5):e8547. doi: 10.15252/emmm.201708547 (PMC6505579; doi:10.15252/emmm.201708547)

AppendixFigS8

AppendixFigS8A_Dnmt1

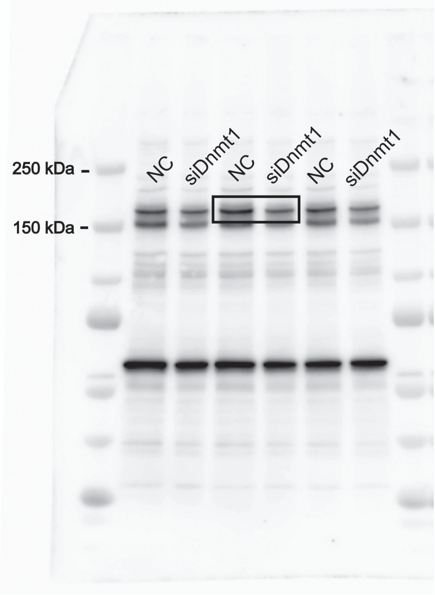

AppendixFigS8A_Gapdh

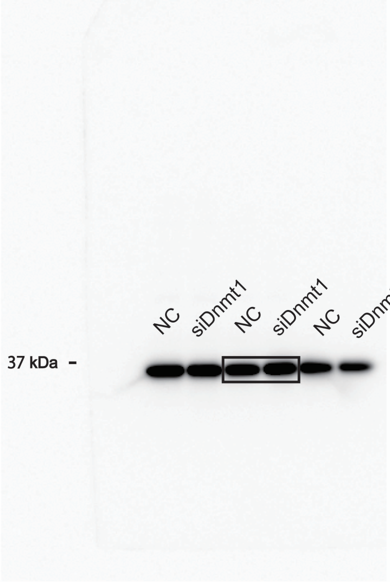

AppendixFigS8B_Dnmt3a

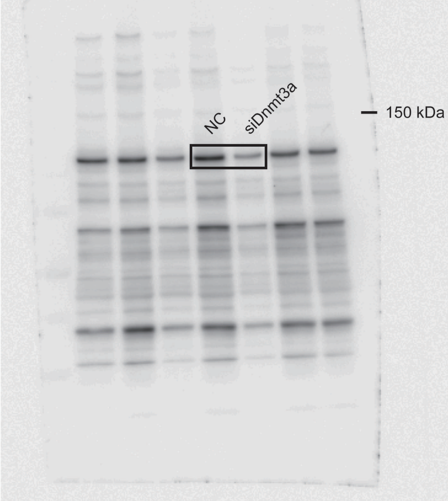

AppendixFigS8B_Gapdh

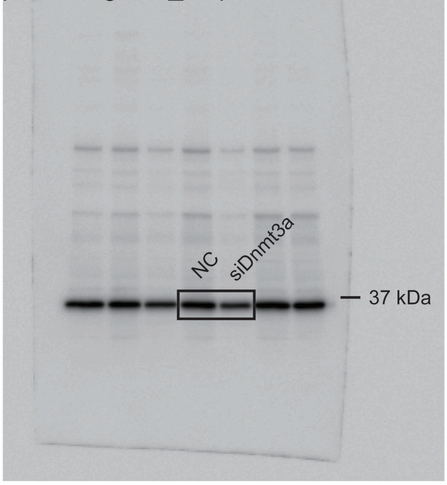

AppendixFigS8C_Dnmt3b

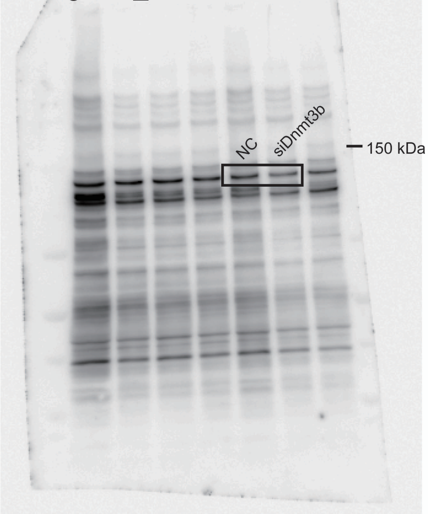

AppendixFigS8C_Gapdh

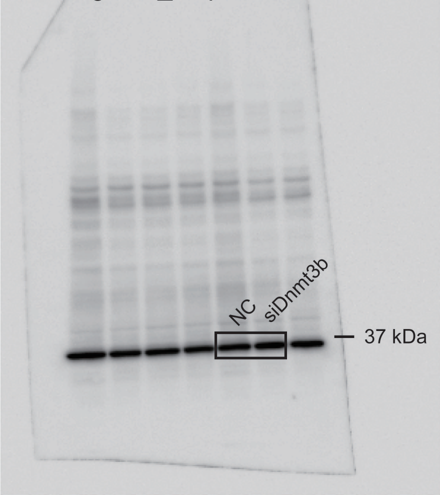

Supplement: Supplementary file 2 — Source Data for Appendix [file EMMM-11-e8547-s010.zip › SourceData_for_Appendix_Figures/SourceData_for_AppendixFigS8_1200DPI.pdf]
