# Supplementary figures and images for: DNA methylation inhibitor attenuates polyglutamine‐induced neurodegeneration by regulating Hes5
Source: EMBO Mol Med. 2019 Apr 1;11(5):e8547. doi: 10.15252/emmm.201708547 (PMC6505579; doi:10.15252/emmm.201708547)

AppendixFigS12\_SH97Q

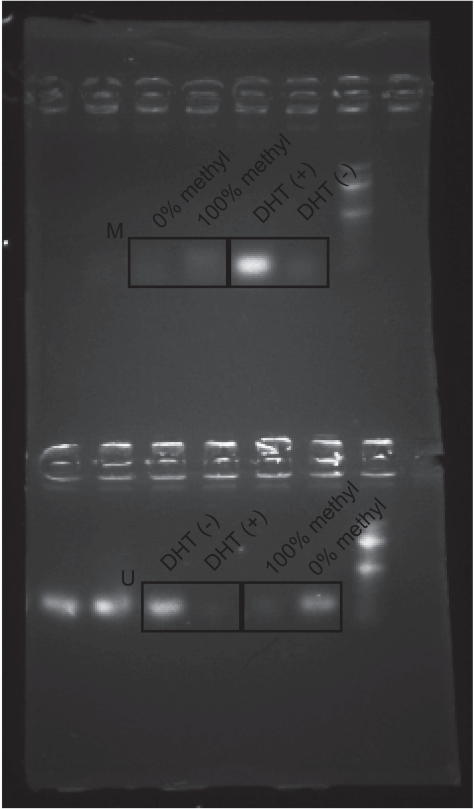

Supplement: Supplementary file 2 — Source Data for Appendix [file EMMM-11-e8547-s010.zip › SourceData_for_Appendix_Figures/SourceData_for_AppendixFigS12_1200DPI.pdf]

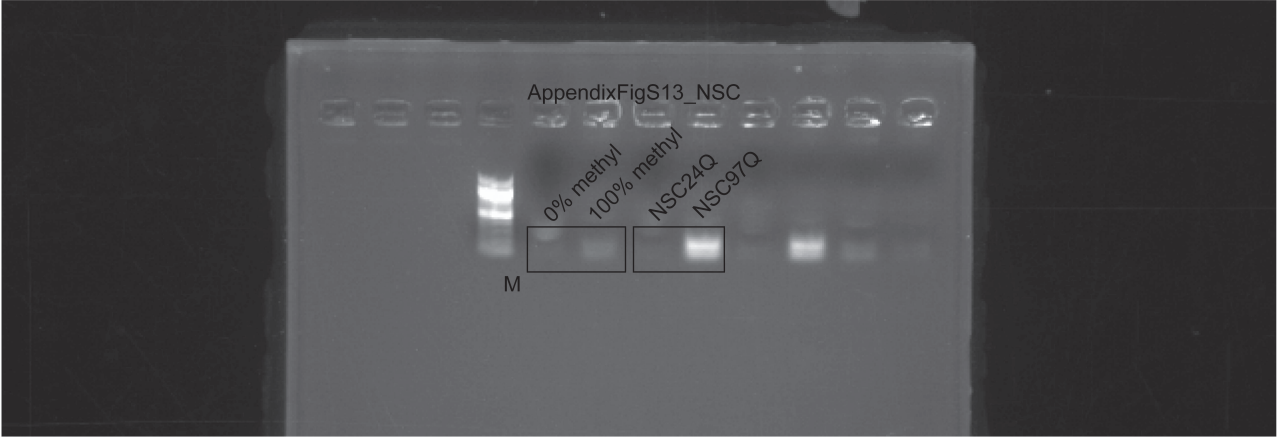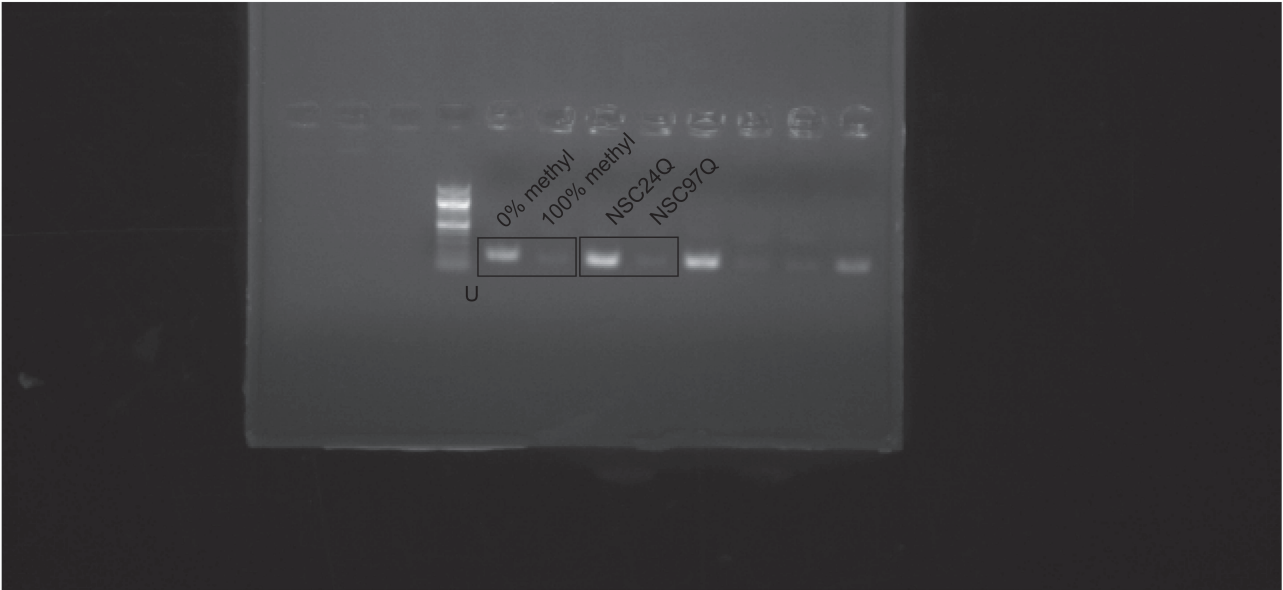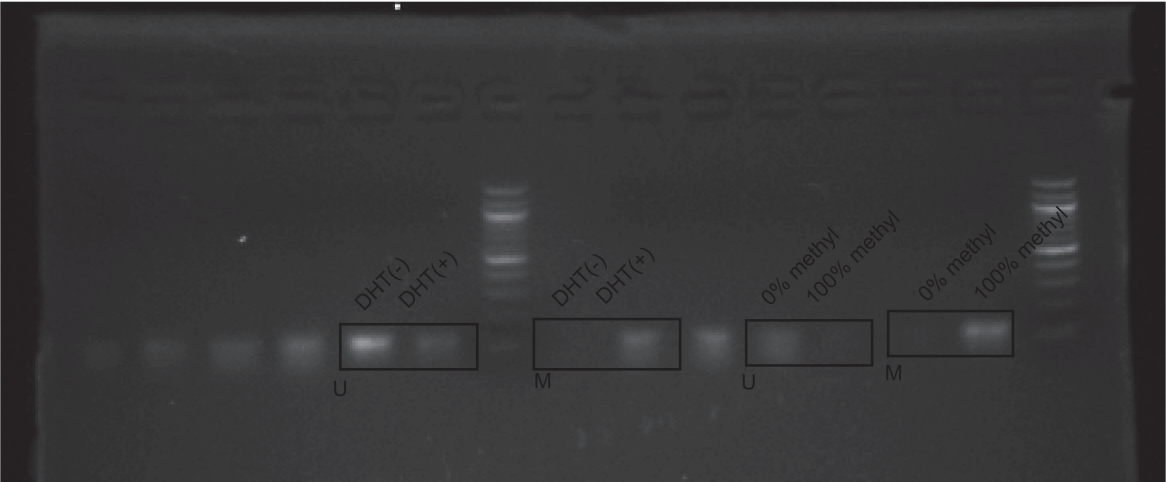

Supplement: Supplementary file 2 — Source Data for Appendix [file EMMM-11-e8547-s010.zip › SourceData_for_Appendix_Figures/SourceData_for_AppendixFigS13_1200DPI.pdf]

## AppendixFigS1

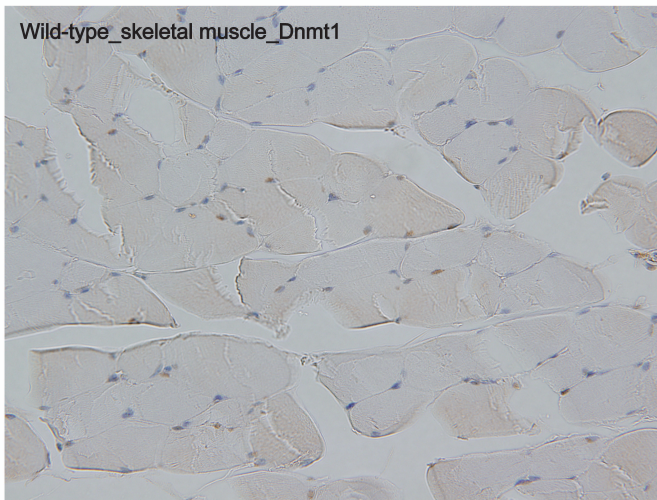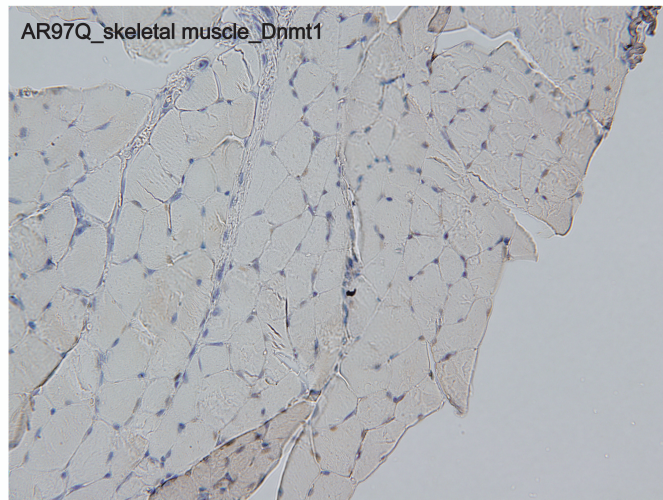

Supplement: Supplementary file 2 — Source Data for Appendix [file EMMM-11-e8547-s010.zip › SourceData_for_Appendix_Figures/SourceData_for_AppendixFigS1_1200DPI.pdf]

## AppendixFigS2

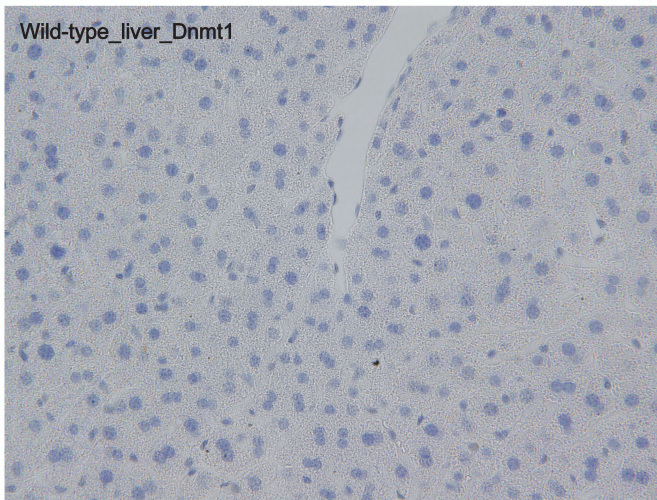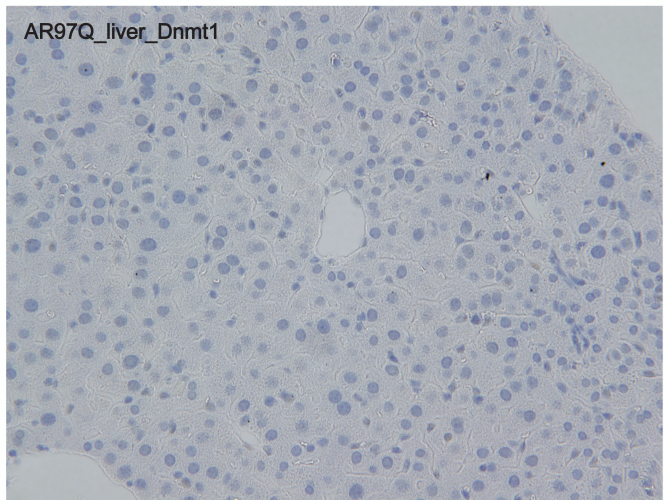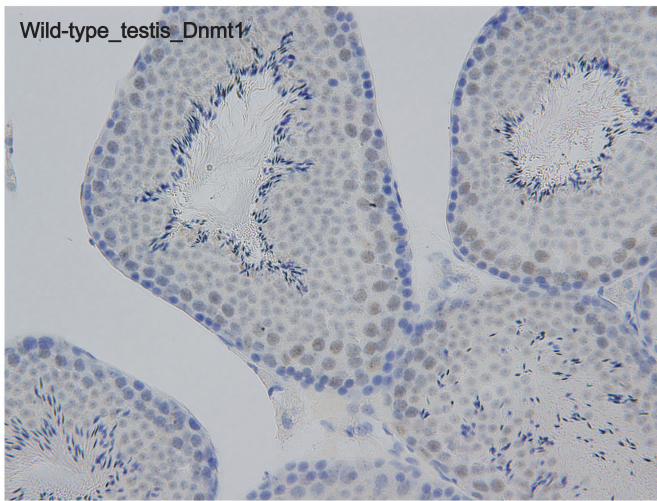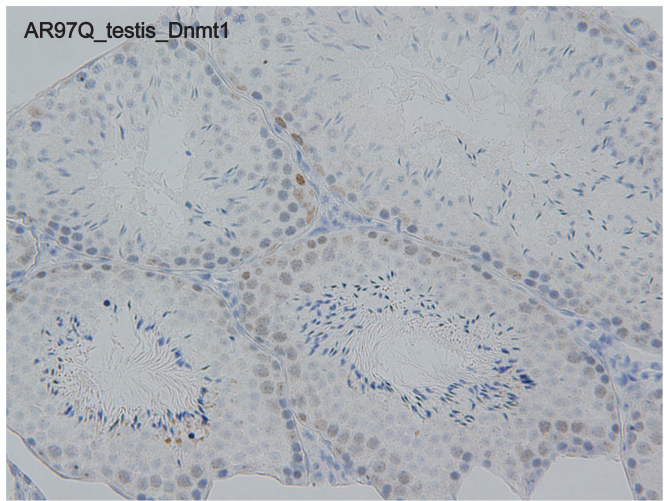

AppendixFigS2

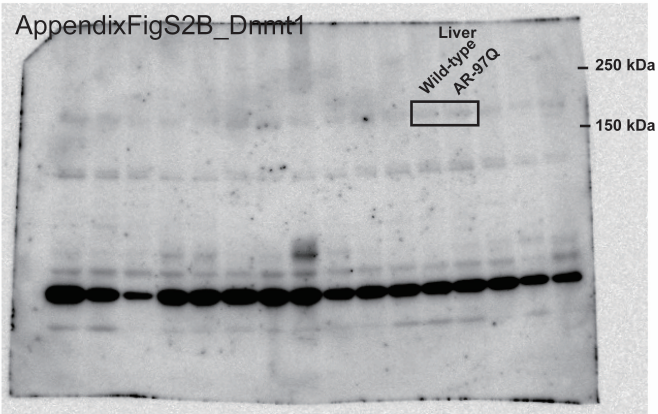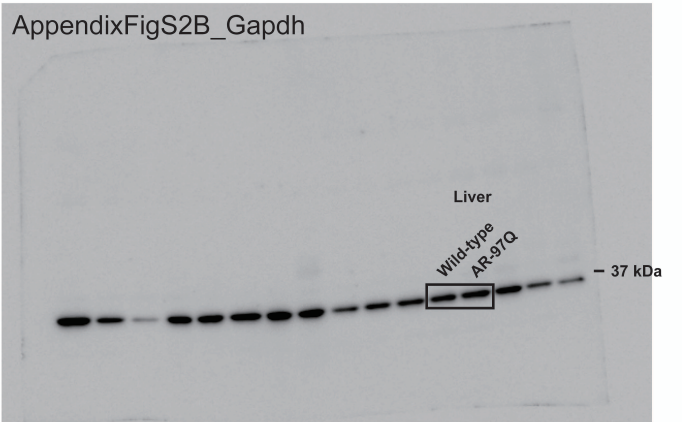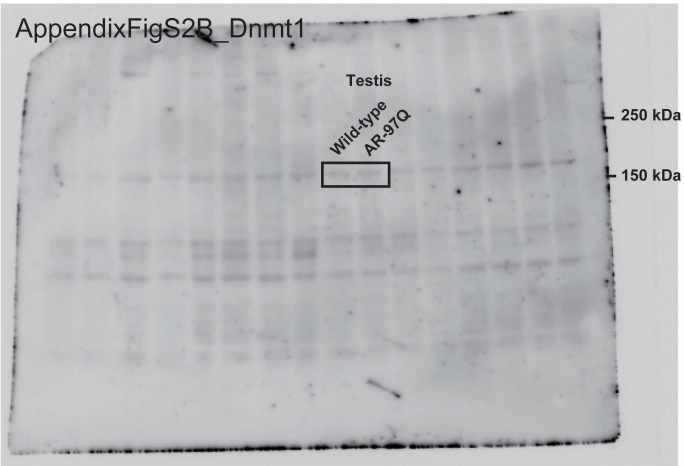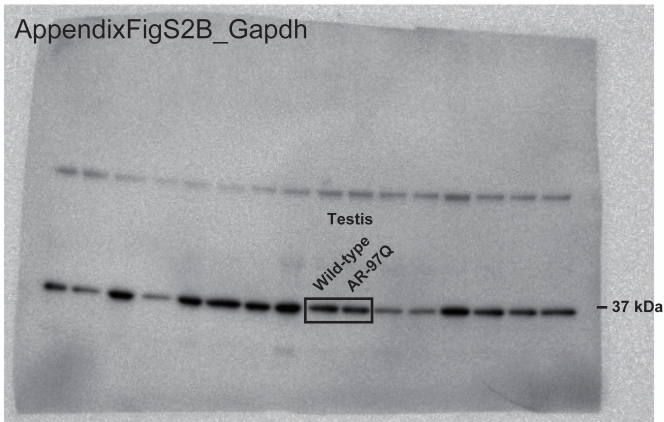

Supplement: Supplementary file 2 — Source Data for Appendix [file EMMM-11-e8547-s010.zip › SourceData_for_Appendix_Figures/SourceData_for_AppendixFigS2_1200DPI.pdf]

## AppendixFigS4

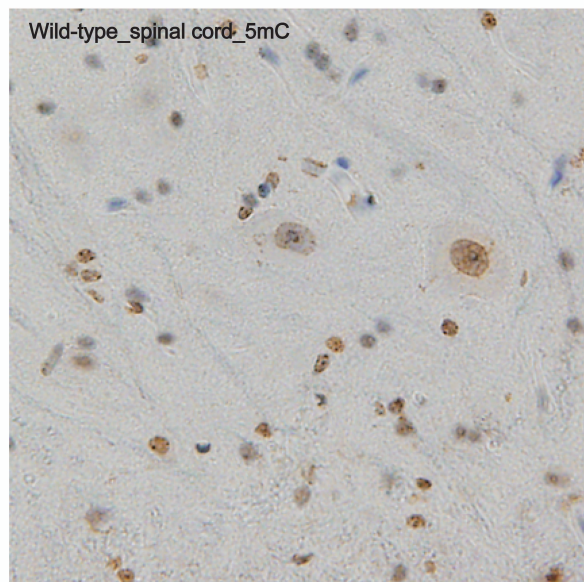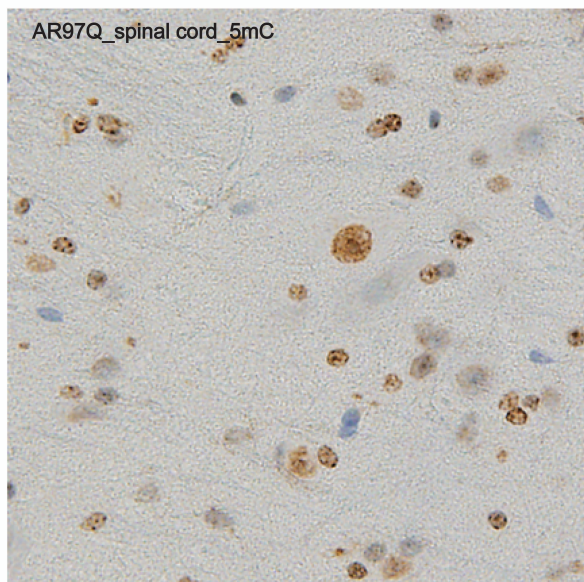

Supplement: Supplementary file 2 — Source Data for Appendix [file EMMM-11-e8547-s010.zip › SourceData_for_Appendix_Figures/SourceData_for_AppendixFigS4_1200DPI.pdf]

## AppendixFigS5

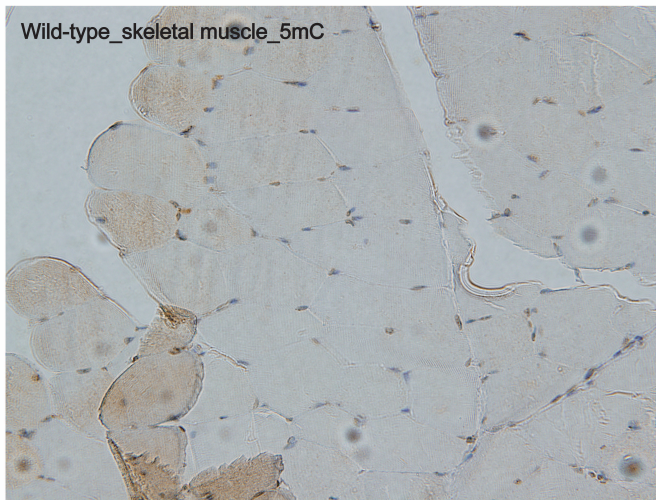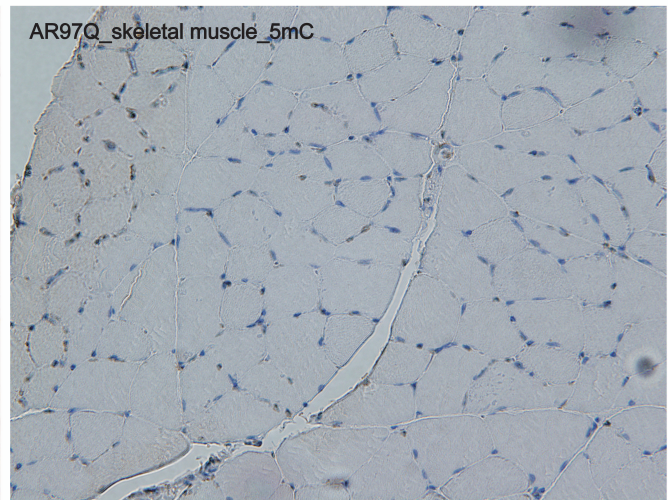

Supplement: Supplementary file 2 — Source Data for Appendix [file EMMM-11-e8547-s010.zip › SourceData_for_Appendix_Figures/SourceData_for_AppendixFigS5_1200DPI.pdf]

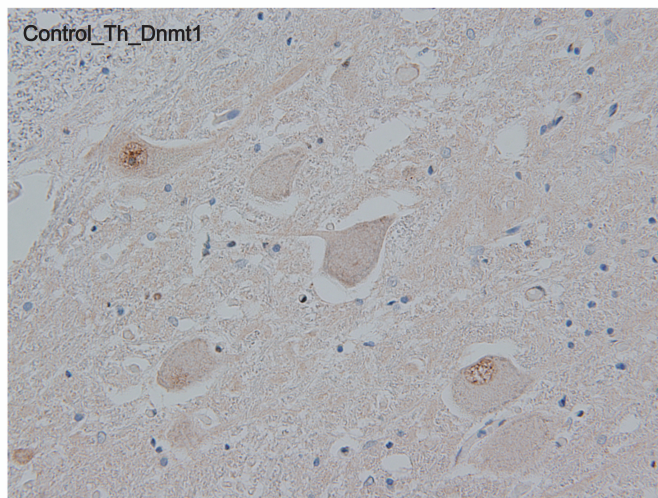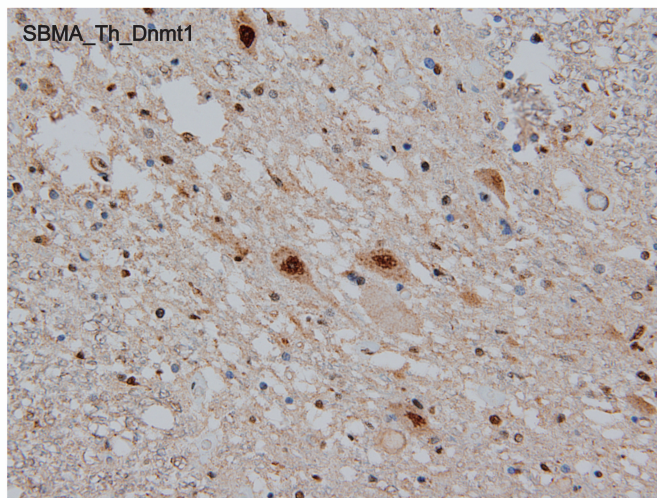

Supplement: Supplementary file 2 — Source Data for Appendix [file EMMM-11-e8547-s010.zip › SourceData_for_Appendix_Figures/SourceData_for_AppendixFigS6_1200DPI.pdf]

Figure1

Fig1A\_Dnmt1

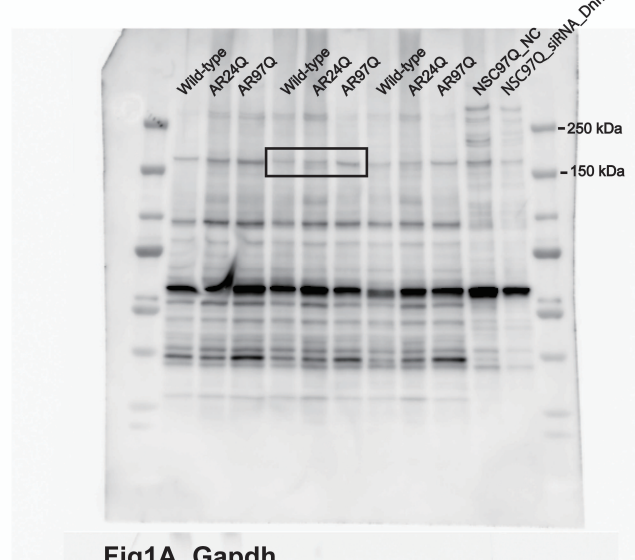

Fig1G\_Dnmt1

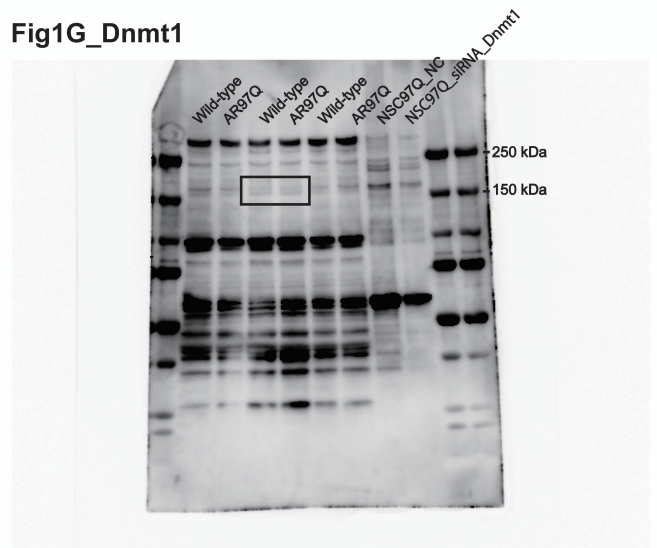

Fig1A\_Gapdh

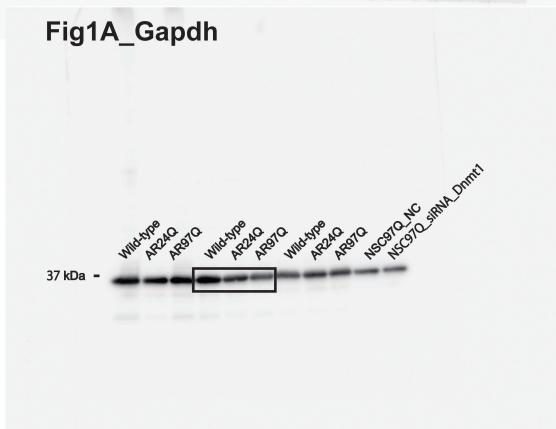

Fig1G\_Gapdh

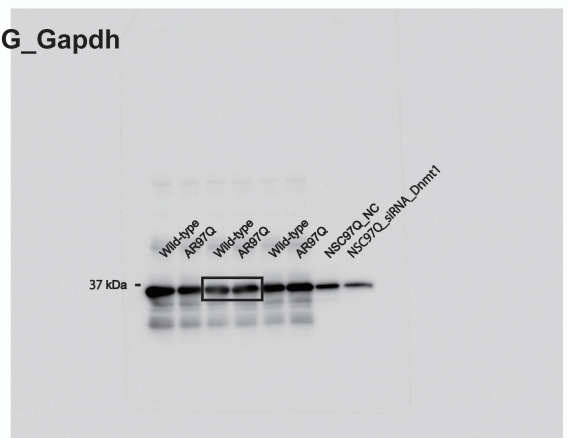

Fig1A\_Dnmt3a

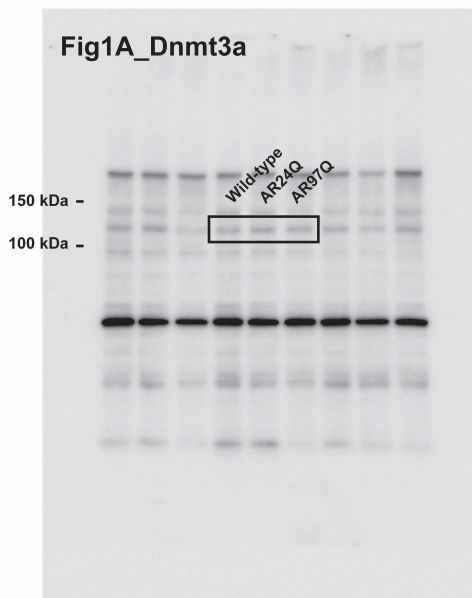

Fig1A\_Dnmt3b

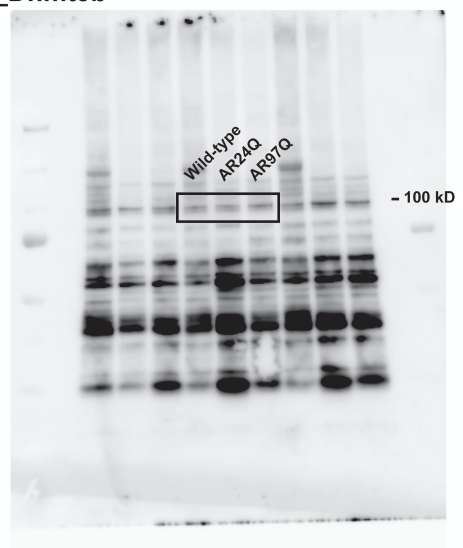

Figure1D

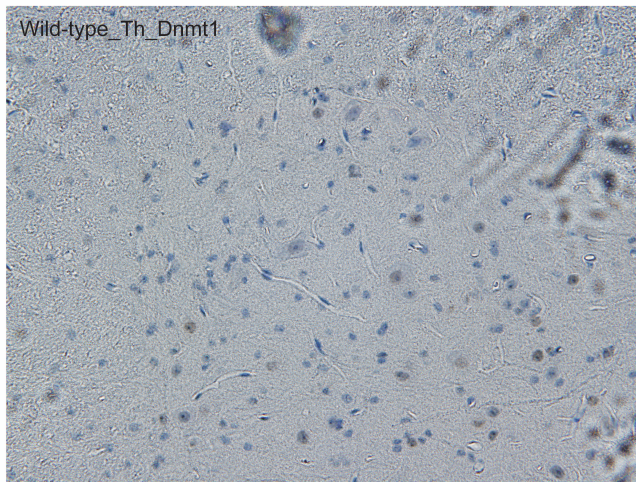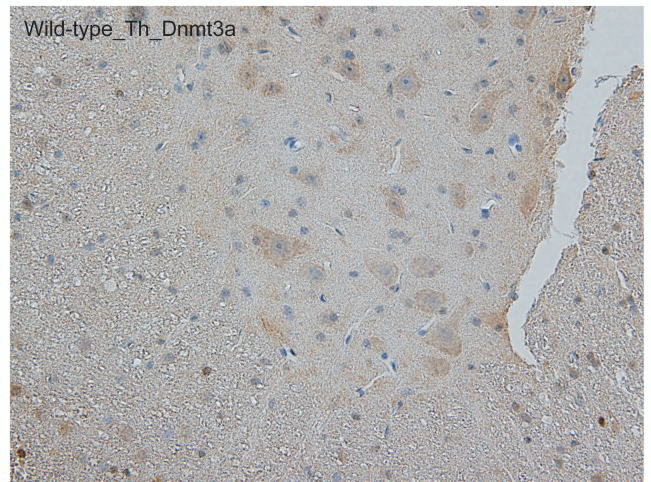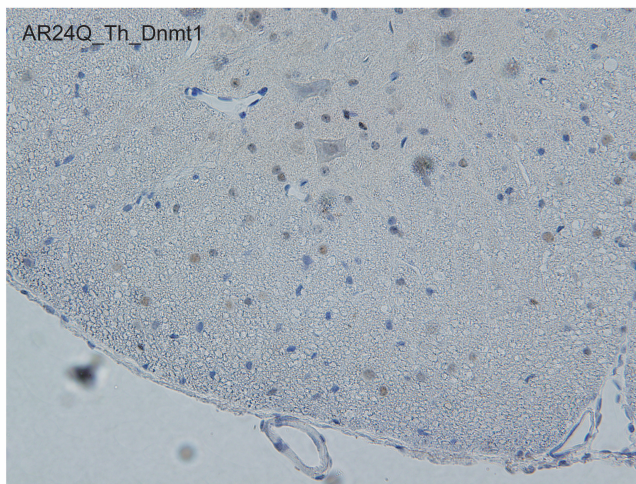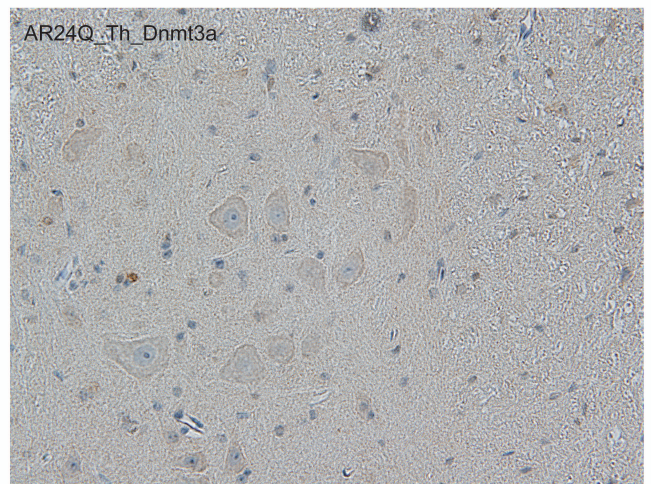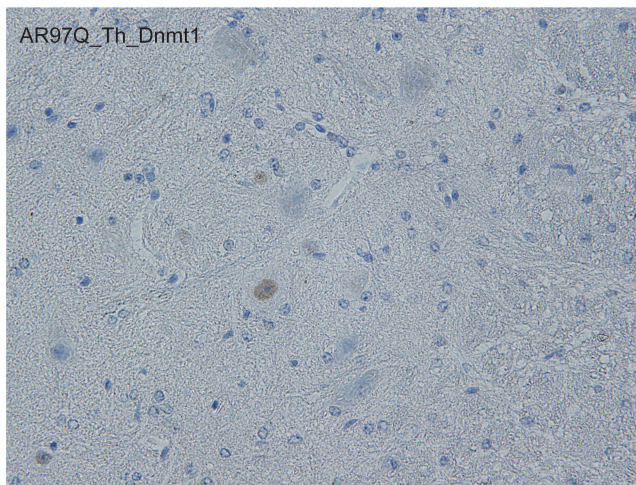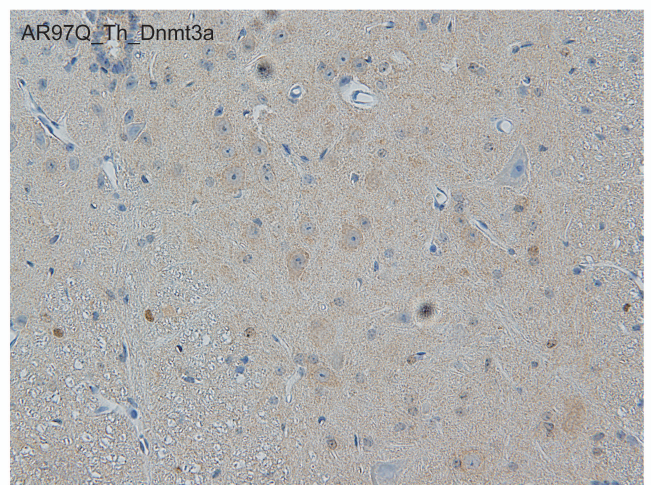

Figure1DF

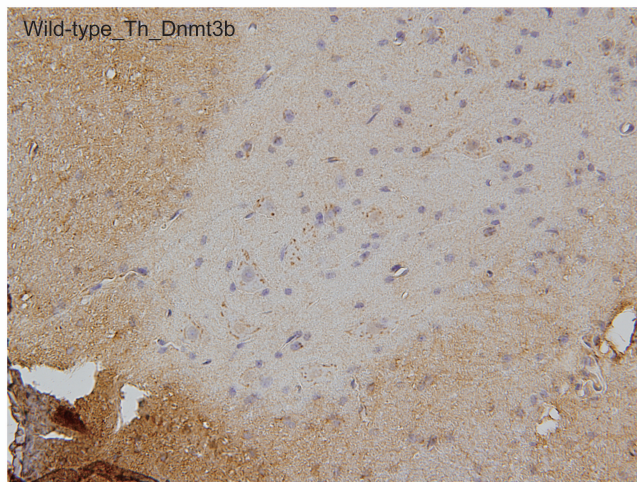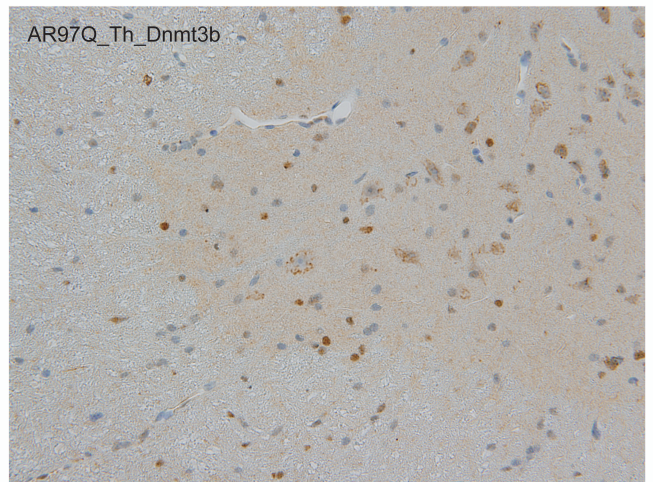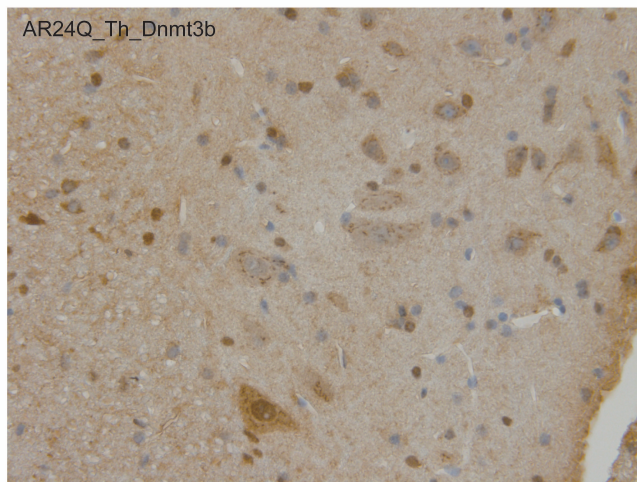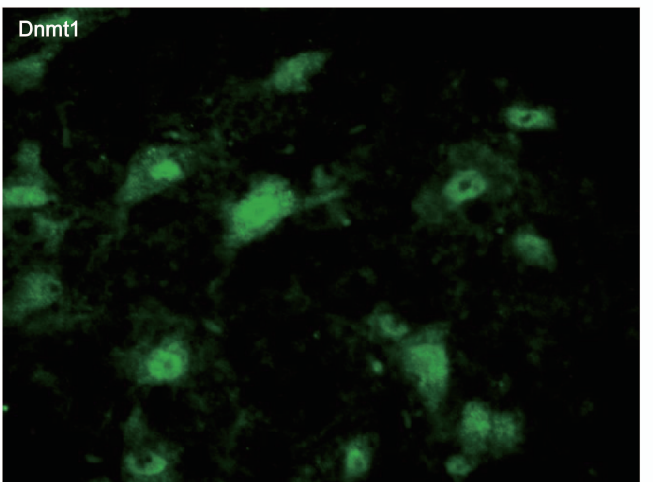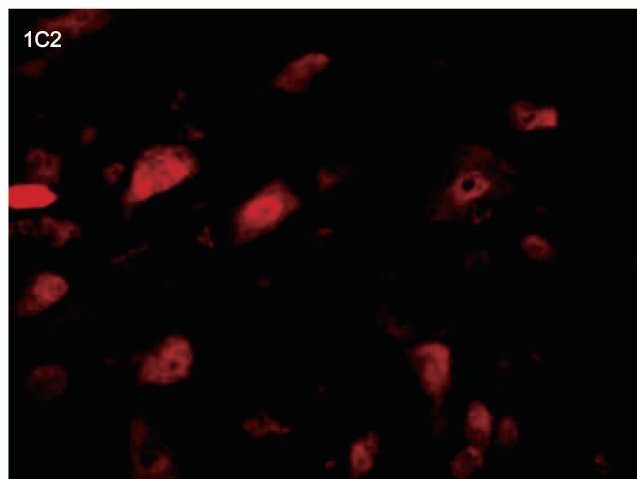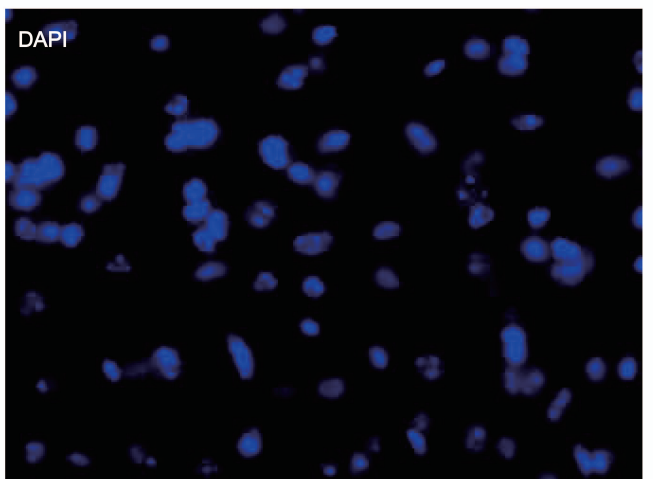

Supplement: Supplementary file 4 — Source Data for Figure 1 [file EMMM-11-e8547-s002.pdf]

Figure3F

Fig3F\_Dnmt1

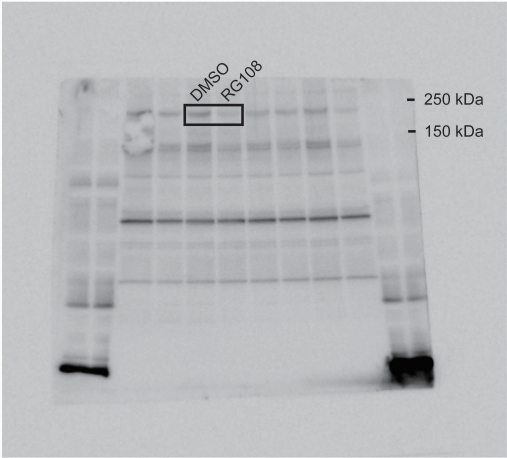

Fig3F\_Gapdh

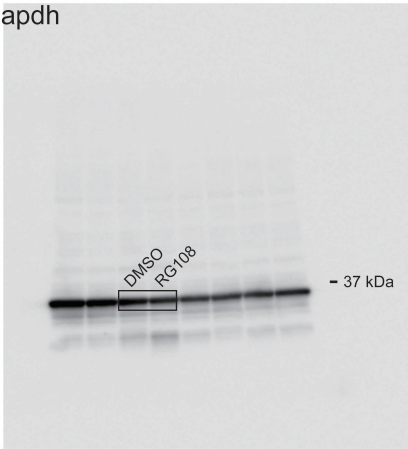

Figure3H

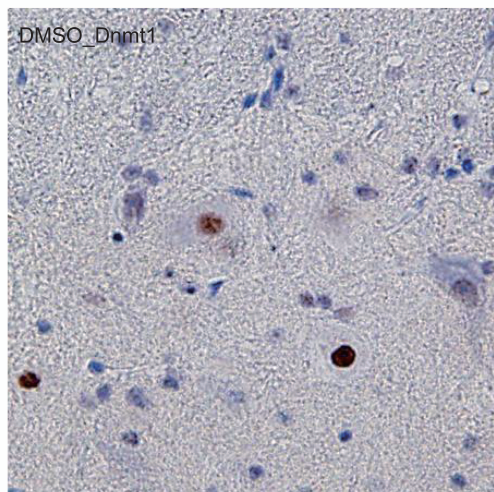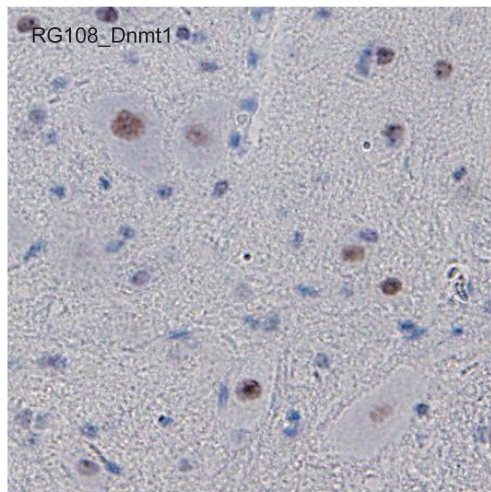

Supplement: Supplementary file 6 — Source Data for Figure 3 [file EMMM-11-e8547-s004.pdf]

Figure4AF

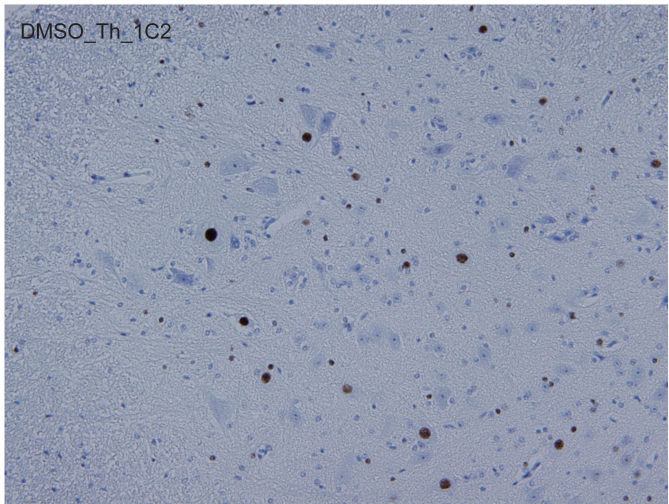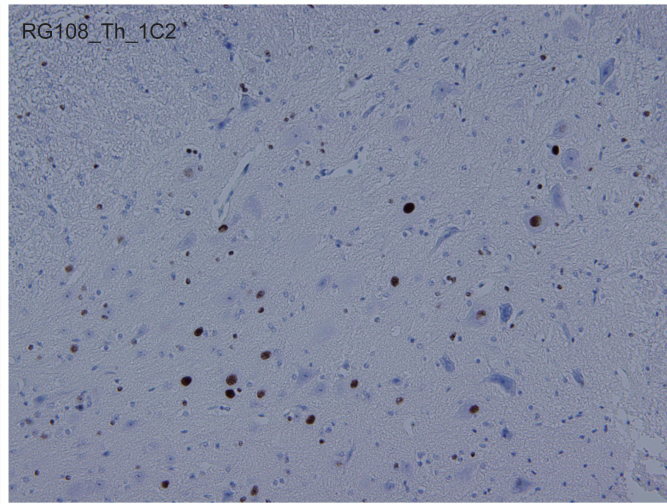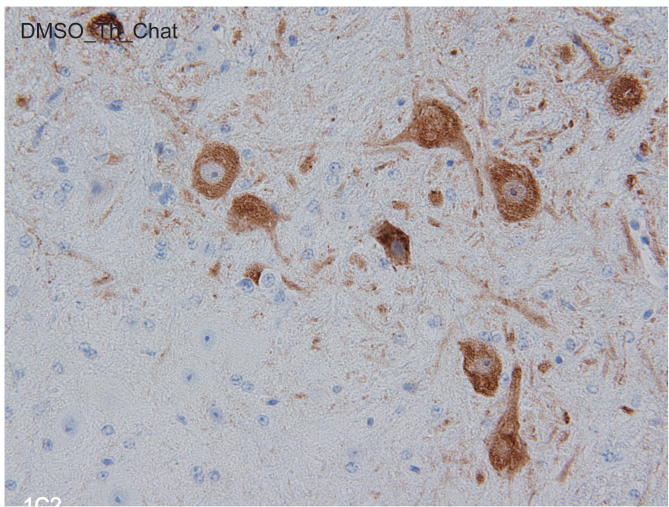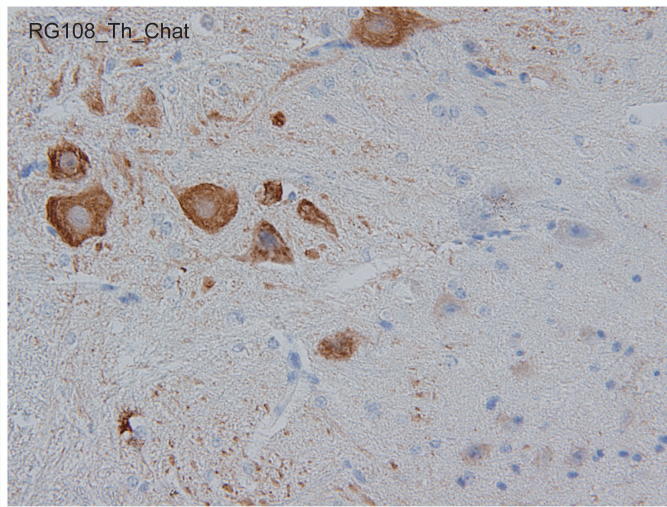

Figure4CH

Fig4C\_AR

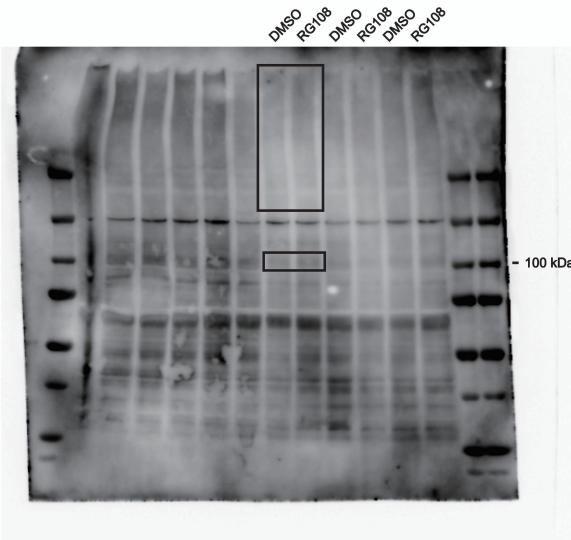

Fig4H\_Chaf

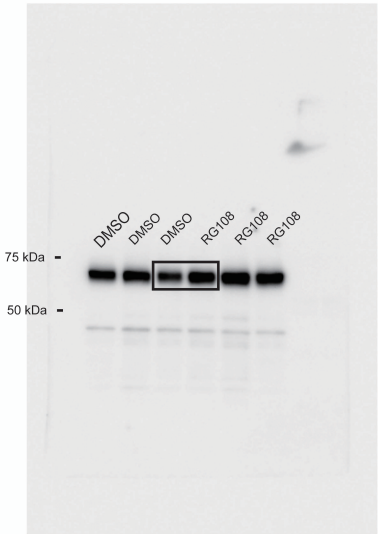

Fig4C\_Gapdh

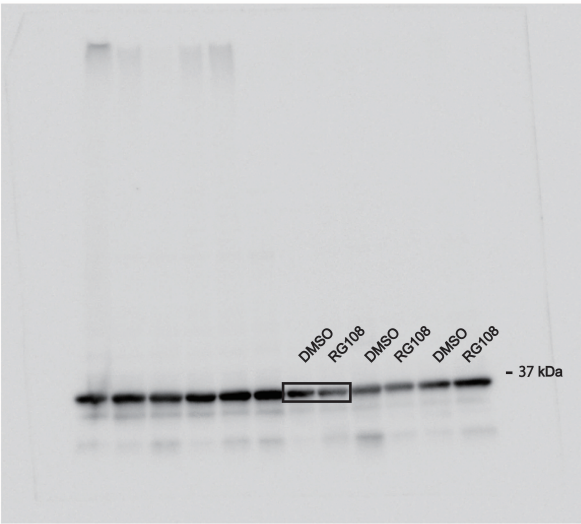

Fig4H\_Gapdh

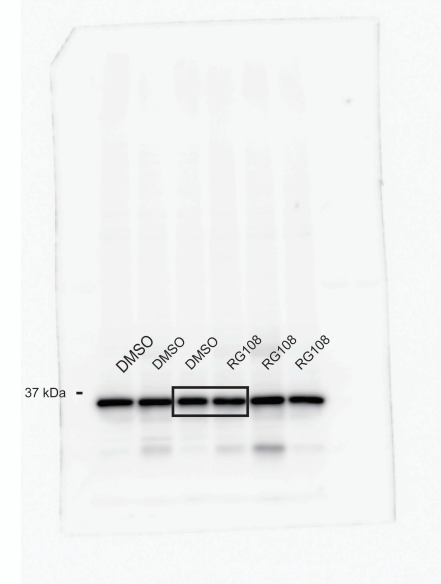

Supplement: Supplementary file 7 — Source Data for Figure 4 [file EMMM-11-e8547-s005.pdf]

Figure6AC

Fig6A\_SH97Q

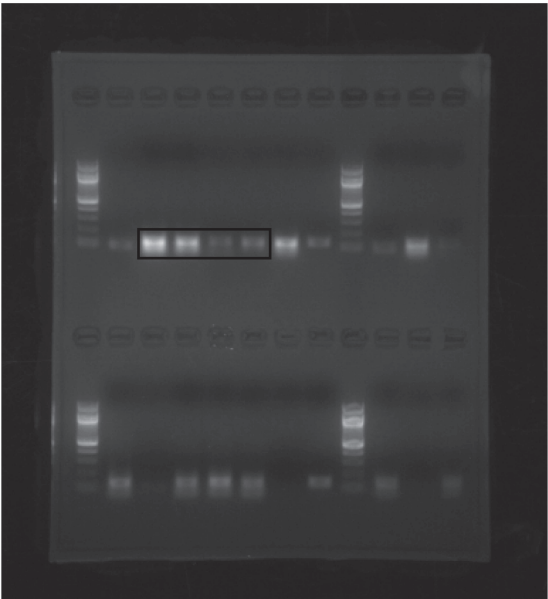

Fig6C\_NSC97Q

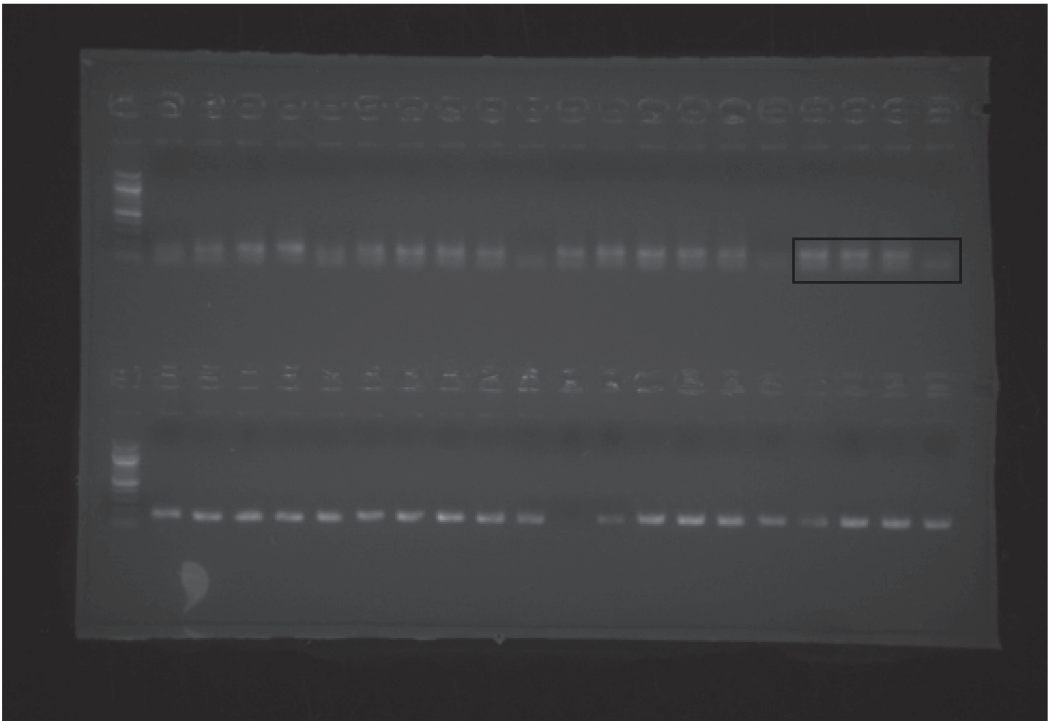

Figure6F

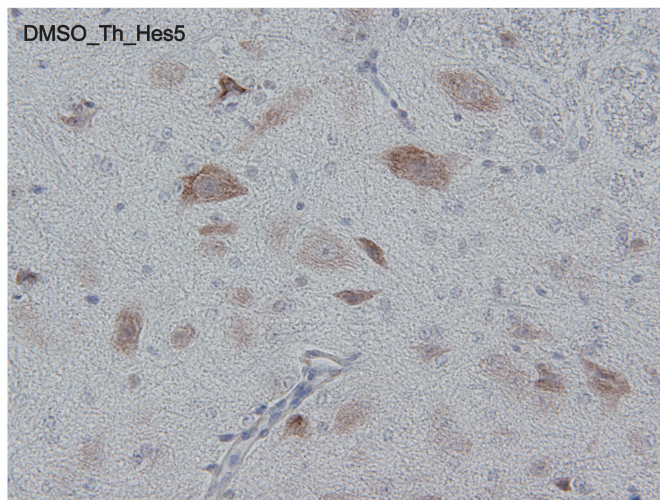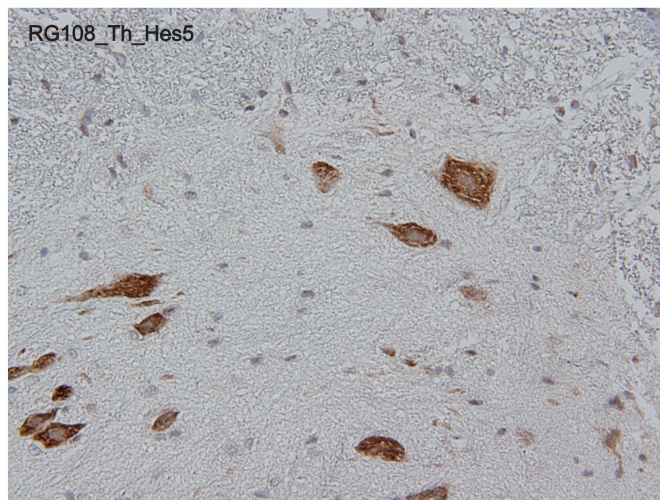

Supplement: Supplementary file 9 — Source Data for Figure 6 [file EMMM-11-e8547-s007.pdf]

Figure7B

Fig7B\_Hes5

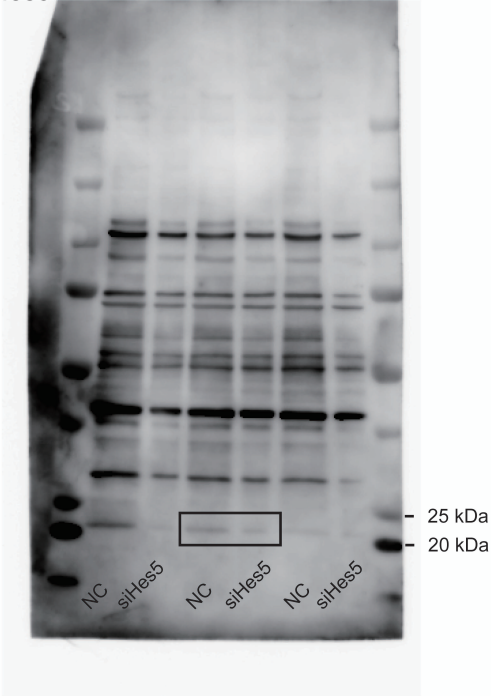

Fig7B\_Gapdh

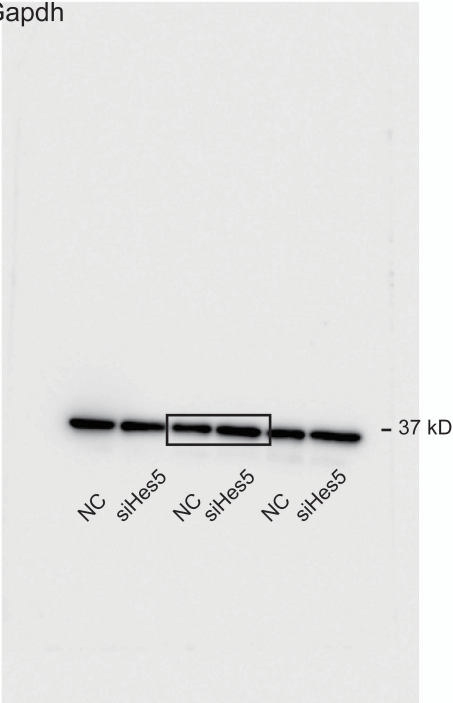

Figure7EG

Fig7E\_Hes5

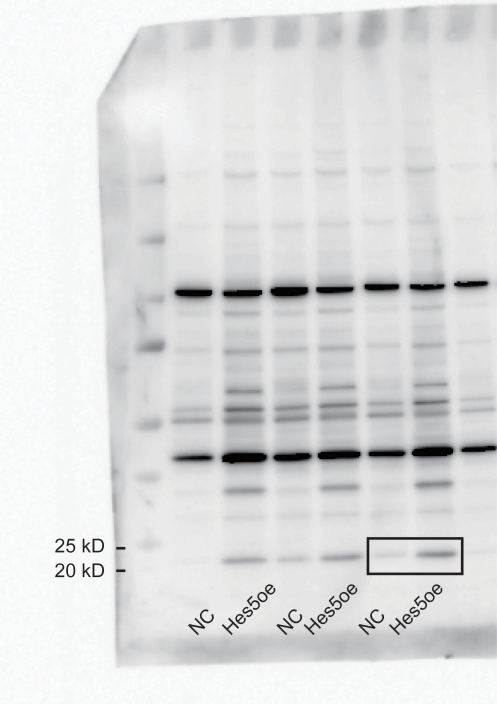

Fig7G\_AR aggregation

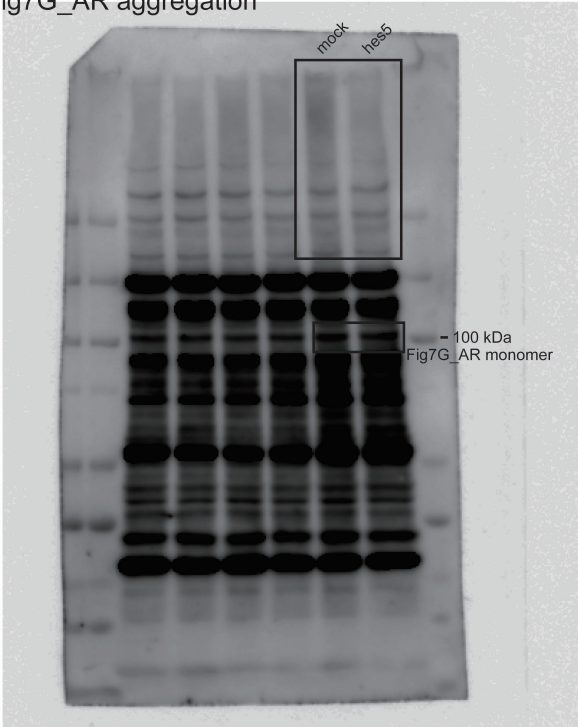

Fig7E\_Gapdh

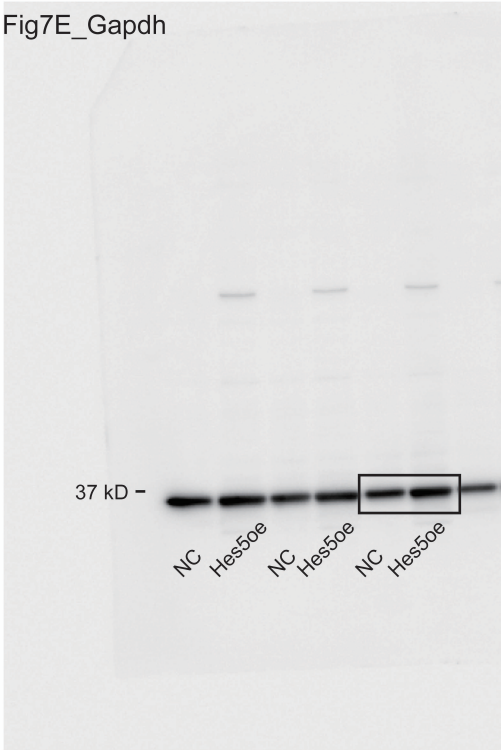

Fig7G\_Gapdh

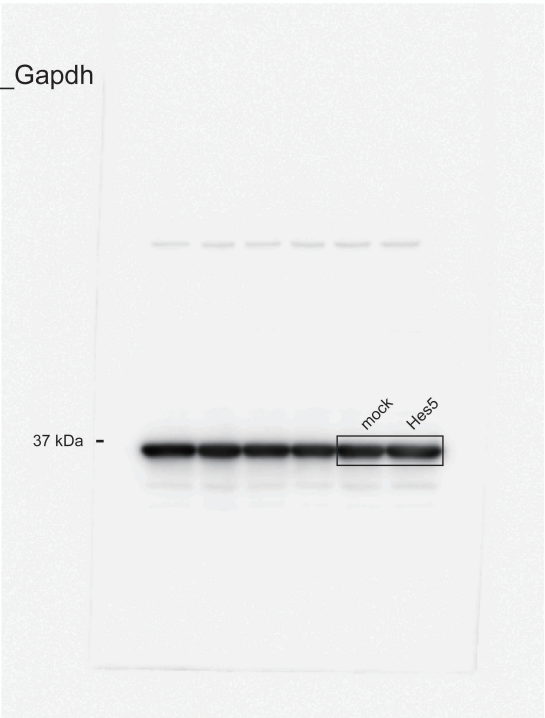

Supplement: Supplementary file 10 — Source Data for Figure 7 [file EMMM-11-e8547-s008.pdf]
