# Supplementary material for: DNA methylation inhibitor attenuates polyglutamine‐induced neurodegeneration by regulating Hes5
Source: EMBO Mol Med. 2019 Apr 1;11(5):e8547. doi: 10.15252/emmm.201708547 (PMC6505579; doi:10.15252/emmm.201708547)

Figure2AC

Fig2A_Dnmt1

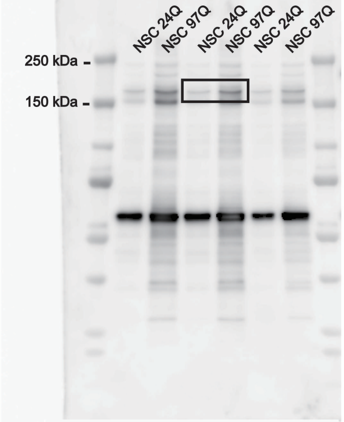

Fig2A_Gapdh

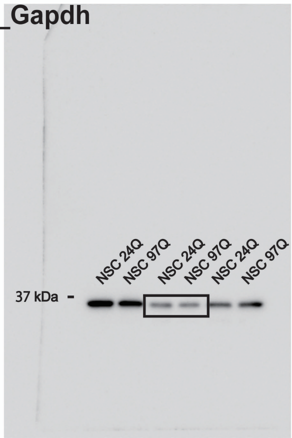

Fig2A_Dnmt3a

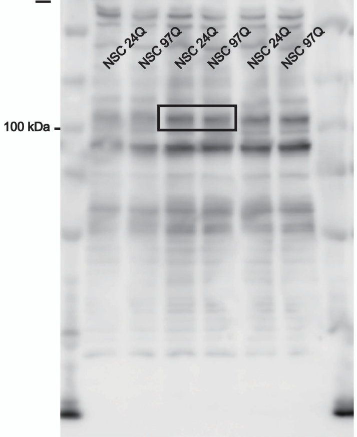

Fig2A_Dnmt3b

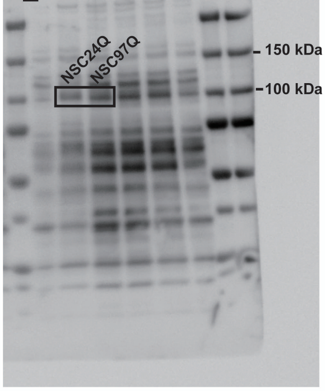

Fig2C_Dnmt1

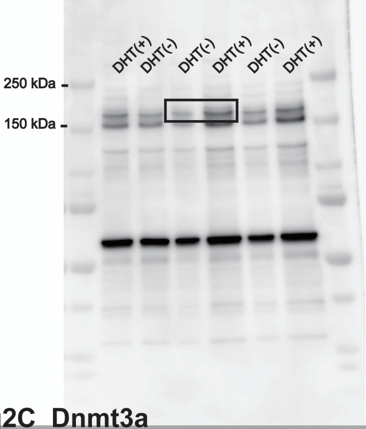

Fig2C_Gapdh

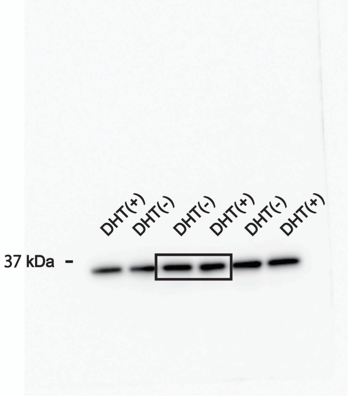

Fig2C_Dnmt3a

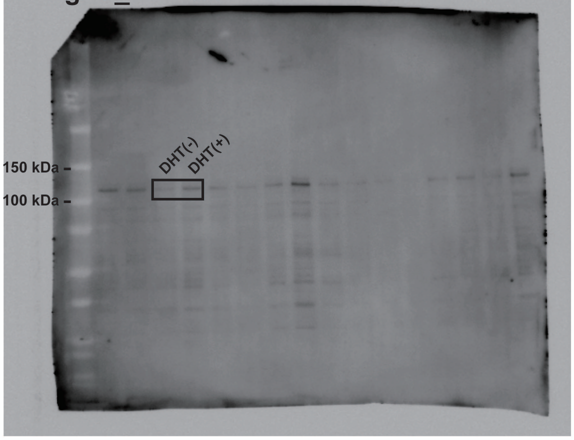

Fig2C_Dnmt3b

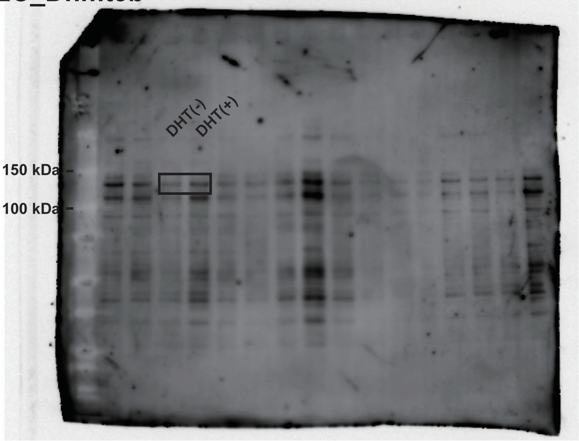

Figure2H

Fig2H_Dnmt1

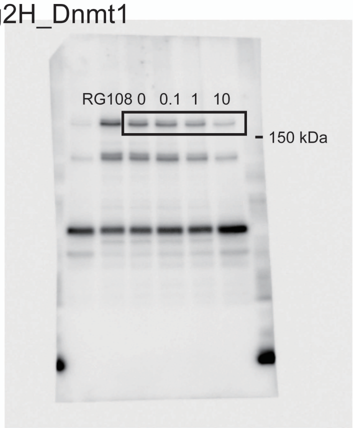

Fig2H_Dnmt3a

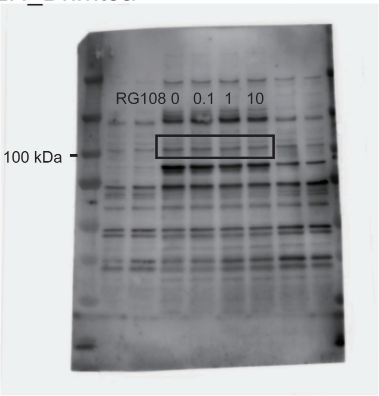

Fig2H_Gapdh

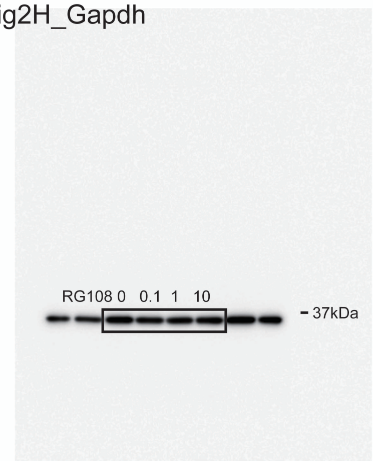

Fig2H_Dnmt3b

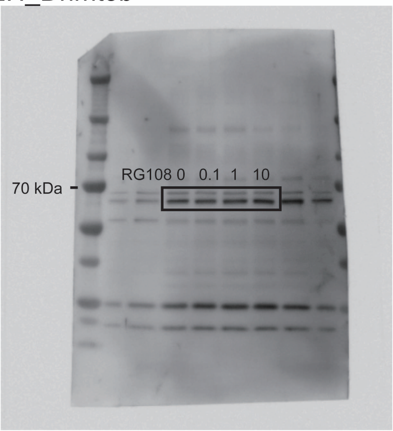

Supplement: Supplementary file 5 — Source Data for Figure 2 [file EMMM-11-e8547-s003.pdf]
