# Supplementary material for: DNA methylation inhibitor attenuates polyglutamine‐induced neurodegeneration by regulating Hes5
Source: EMBO Mol Med. 2019 Apr 1;11(5):e8547. doi: 10.15252/emmm.201708547 (PMC6505579; doi:10.15252/emmm.201708547)

Figure5A

Fig5A_Dnmt1

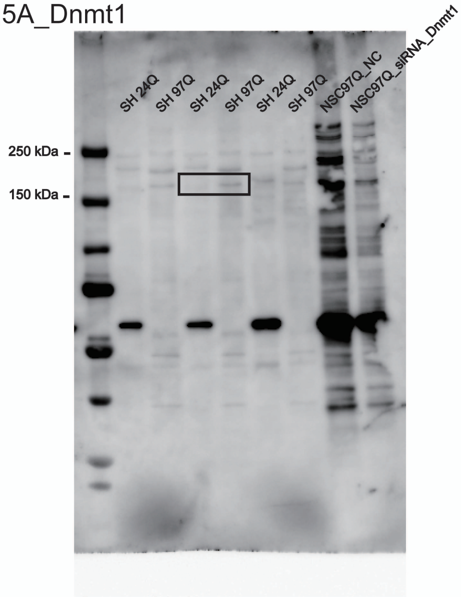

Fig5A_Dnmt3b

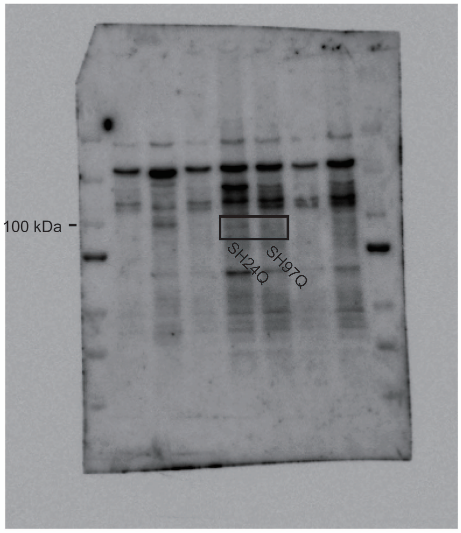

Fig5A_Dnmt3a

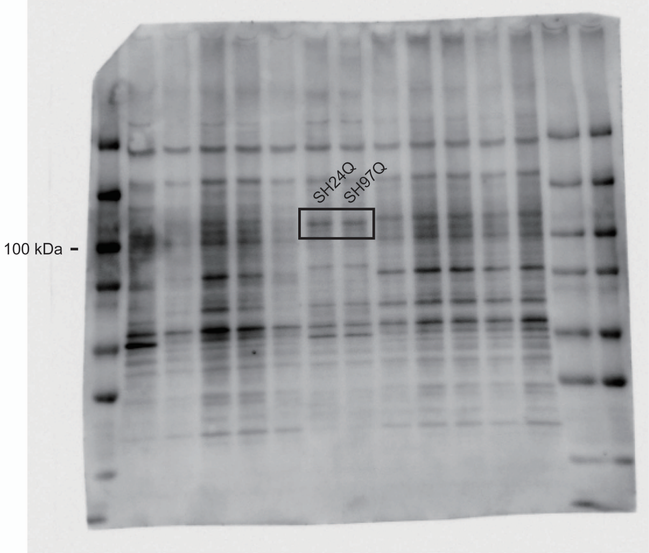

Fig5A_GAPDH

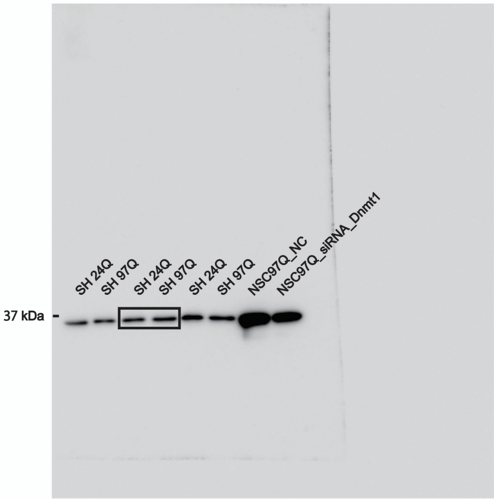

Figure5CH

Fig5C_Dnmt1

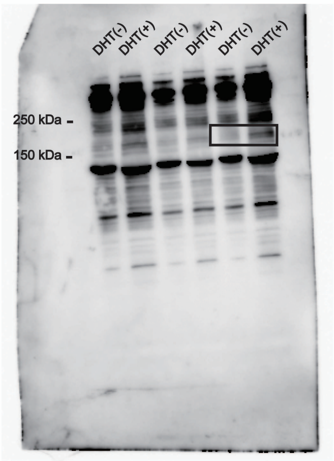

Fig5C_Dnmt3a

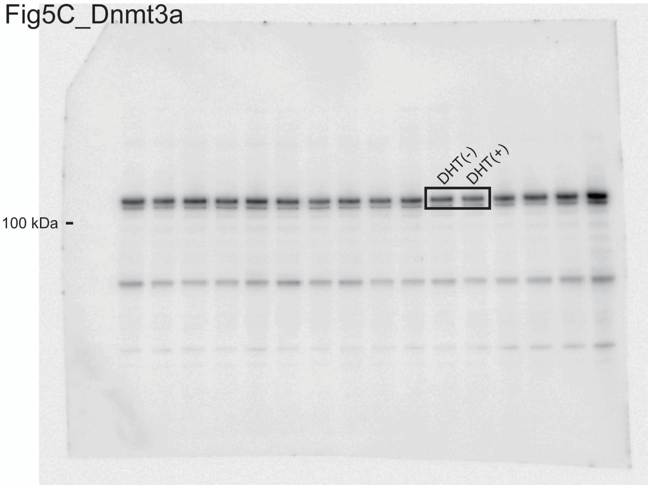

Fig5C_GAPDH

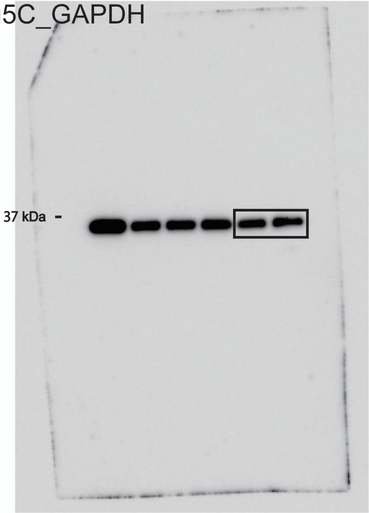

Fig5C_Dnmt3b

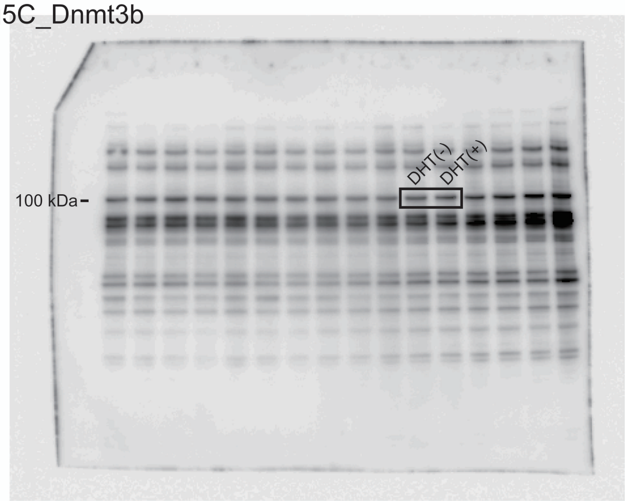

Fig5H

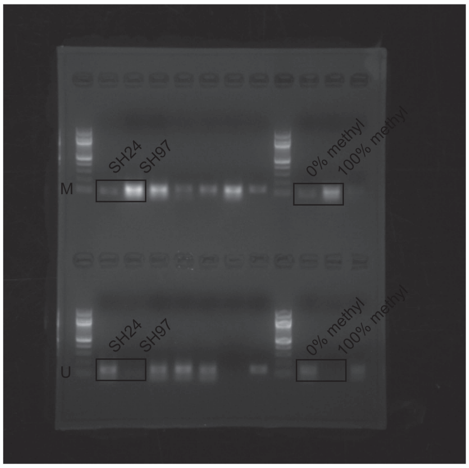

Figure5I

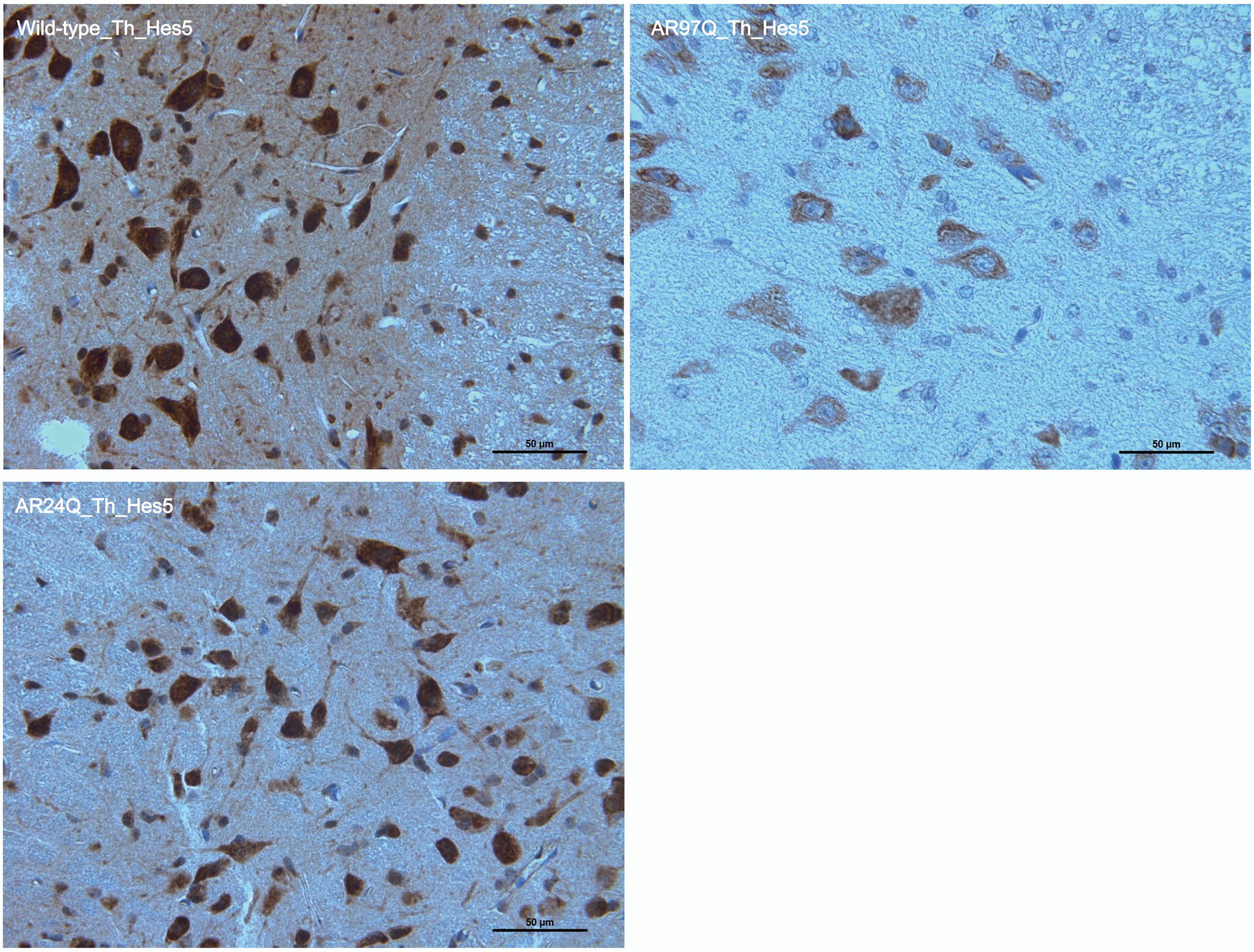

Supplement: Supplementary file 8 — Source Data for Figure 5 [file EMMM-11-e8547-s006.pdf]
