# Supplementary material for: DNA methylation inhibitor attenuates polyglutamine‐induced neurodegeneration by regulating Hes5
Source: EMBO Mol Med. 2019 Apr 1;11(5):e8547. doi: 10.15252/emmm.201708547 (PMC6505579; doi:10.15252/emmm.201708547)

Figure8A

Fig8A_pSmad2

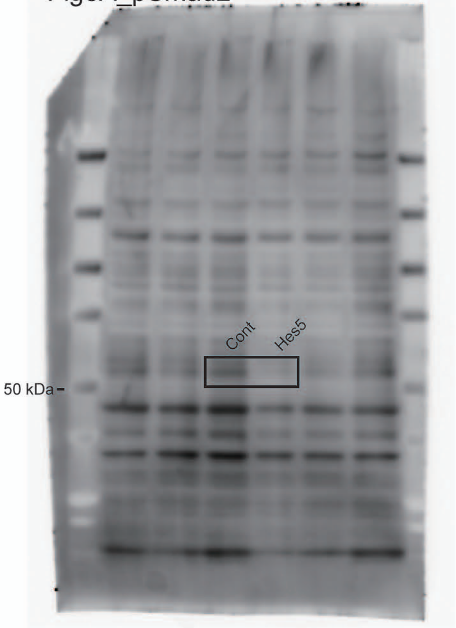

Fig8A_pIkBα

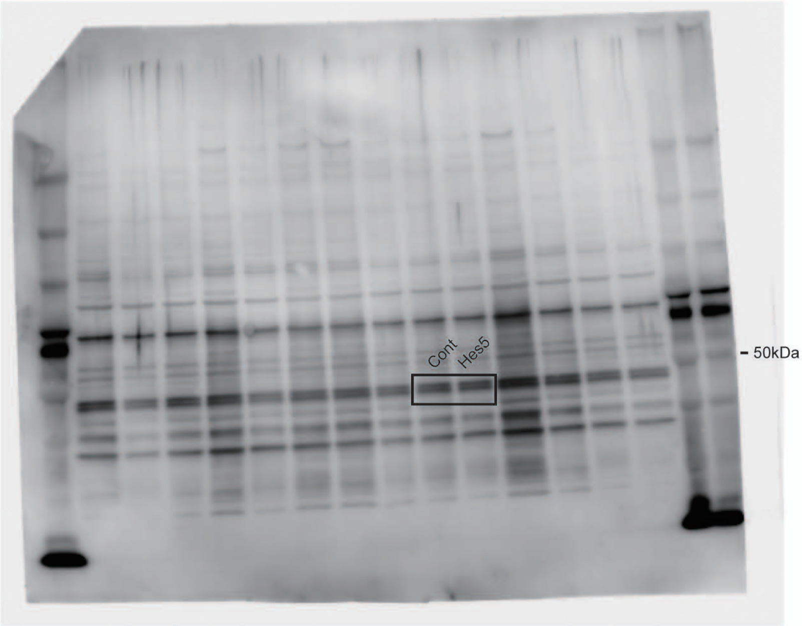

Fig8A_Smad2

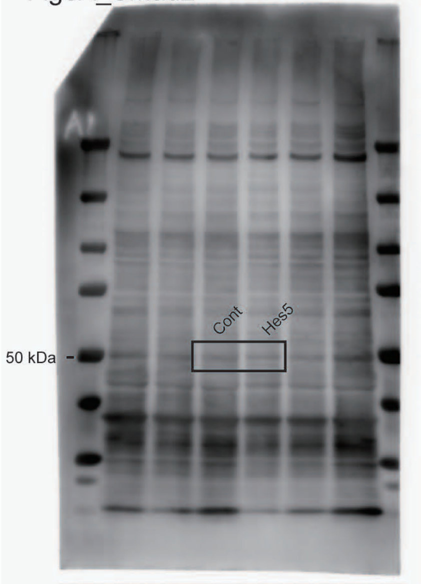

Fig8A_Hsf1

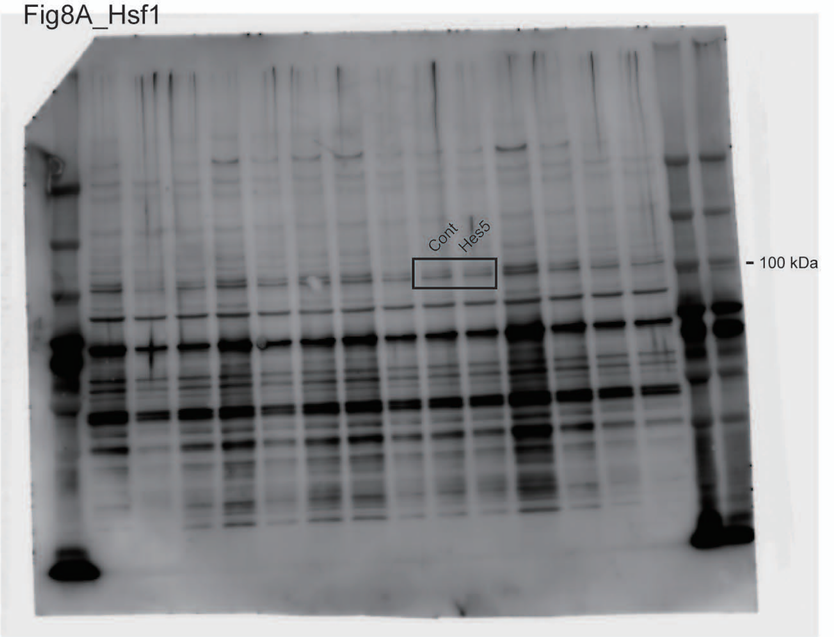

Fig8A_Gapdh

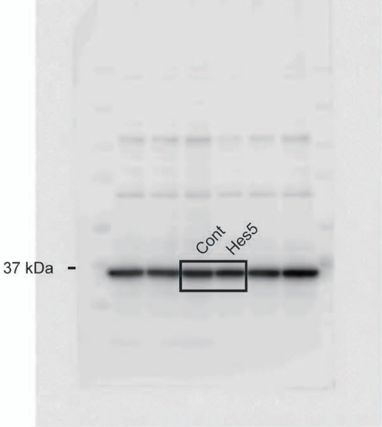

Figure8CD

Fig8C_Hes5

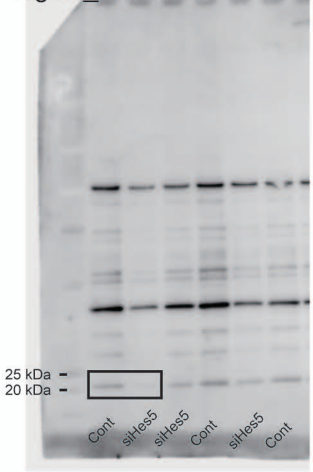

Fig8D_Smad2

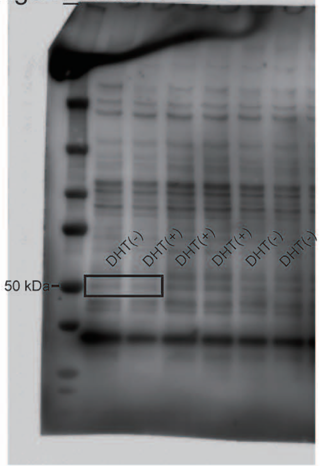

Fig8C_Gapdh

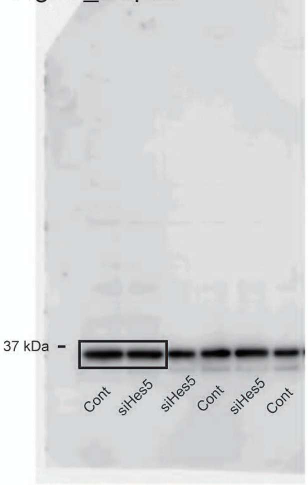

Fig8D_Gapdh

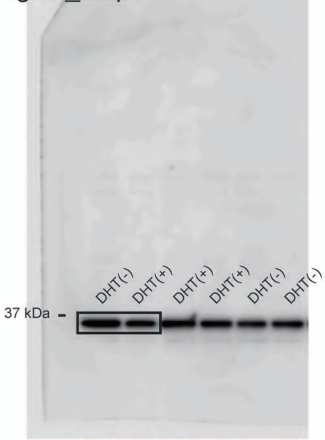

Fig8D_pSmad2

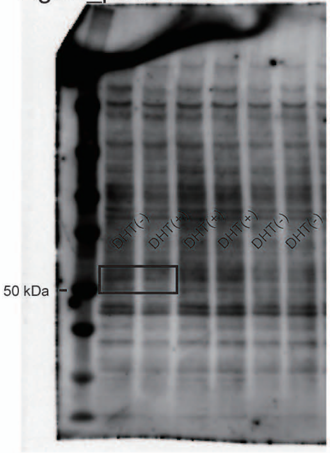

Figure8EF

Fig8E_pSmad2

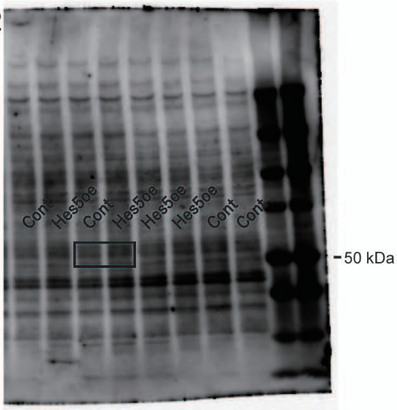

Fig8E_Smad2

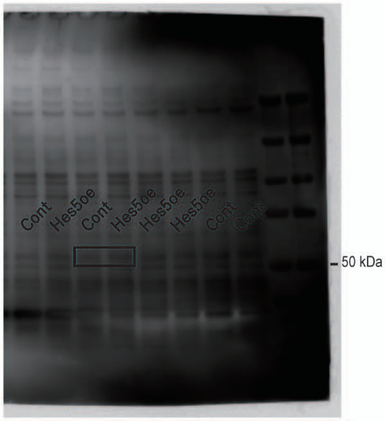

Fig8E_Gapdh

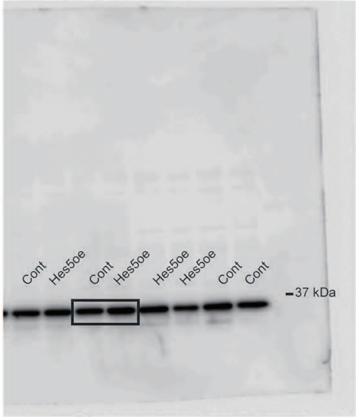

Fig8E_Hes5

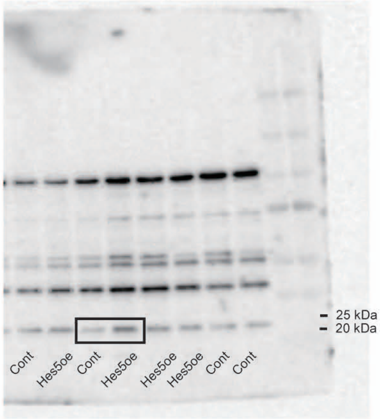

Fig8F_pSmad2

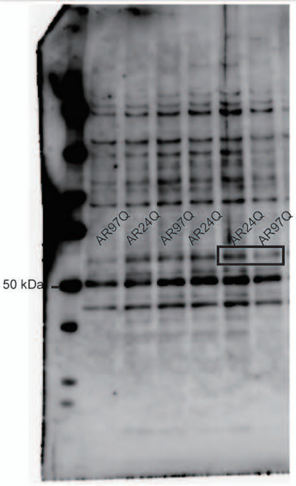

Fig8F_Smad2

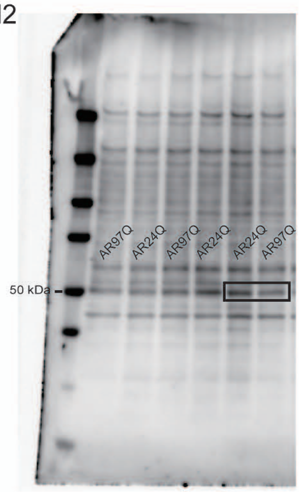

Fig8F_Gapdh

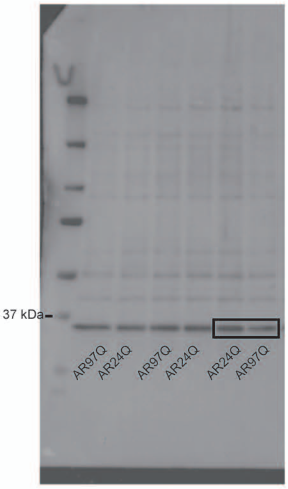

Fig8F_Hes5

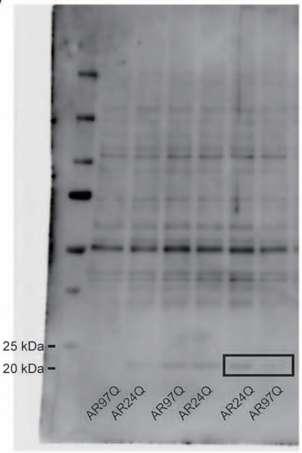

Figure8I

Fig8I_Hes5

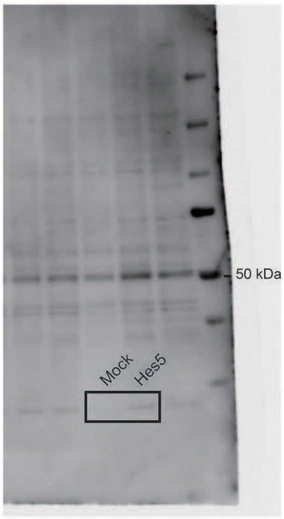

Fig8I_Smad2

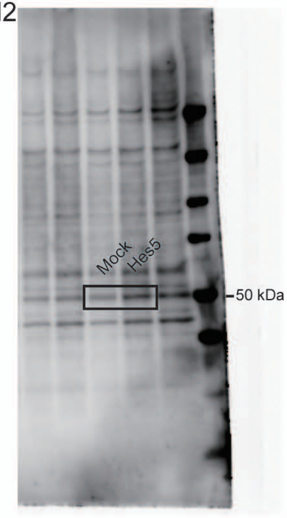

Fig8I_pSmad2

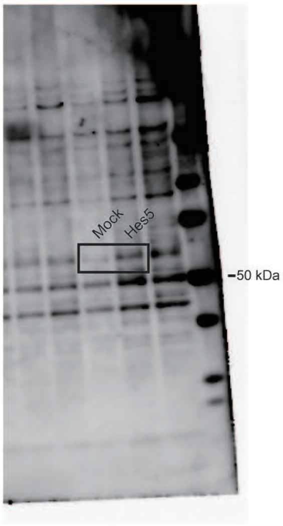

Fig8I_Gapdh

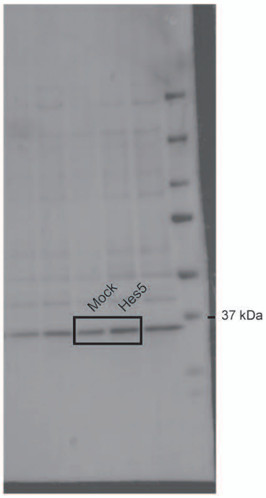

Supplement: Supplementary file 11 — Source Data for Figure 8 [file EMMM-11-e8547-s009.pdf]
